# Supplementary material for: Concurrent trajectories of part-time work and sickness absence: a longitudinal cohort study over 11 years among shift working hospital employees
Source: BMJ Open. 2023 Sep 25;13(9):e072987. doi: 10.1136/bmjopen-2023-072987 (PMC10533668; doi:10.1136/bmjopen-2023-072987)
Supplement: Supplementary data [file bmjopen-2023-072987supp001.pdf]

Supplemental material

Concurrent trajectories of part-time work and sickness absence - a longitudinal cohort study over 11 years among shift working hospital employees

Supplemental material BMJ Publishing Group Limited (BMJ) disclaims all liability and responsibility arising from any reliance placed on this supplemental material which has been supplied by the author(s) BMJ Open

Supplemental material

Supplemental Table S1 Goodness of fit statistics of group-based trajectory analysis models

	Smallest group				
	N	%	BIC	AIC	APP
2-cluster model	5461	17	-263123.4	-263077.8	0.92
3-cluster model	5184	16	-254453.0	-254382.6	0.91
**4-cluster model***	**1671**	**5**	**-250141.8**	**-250046.5**	**0.91**
5-cluster model	919	4	-248427.7	-248307.6	0.84
6-cluster model	920	4	-244756.5	-244611.4	0.83

* The models presented are shown in bold. BIC = Bayesian Information Criterion, AIC = Akaike Information Criterion, and APP = average posterior probability.

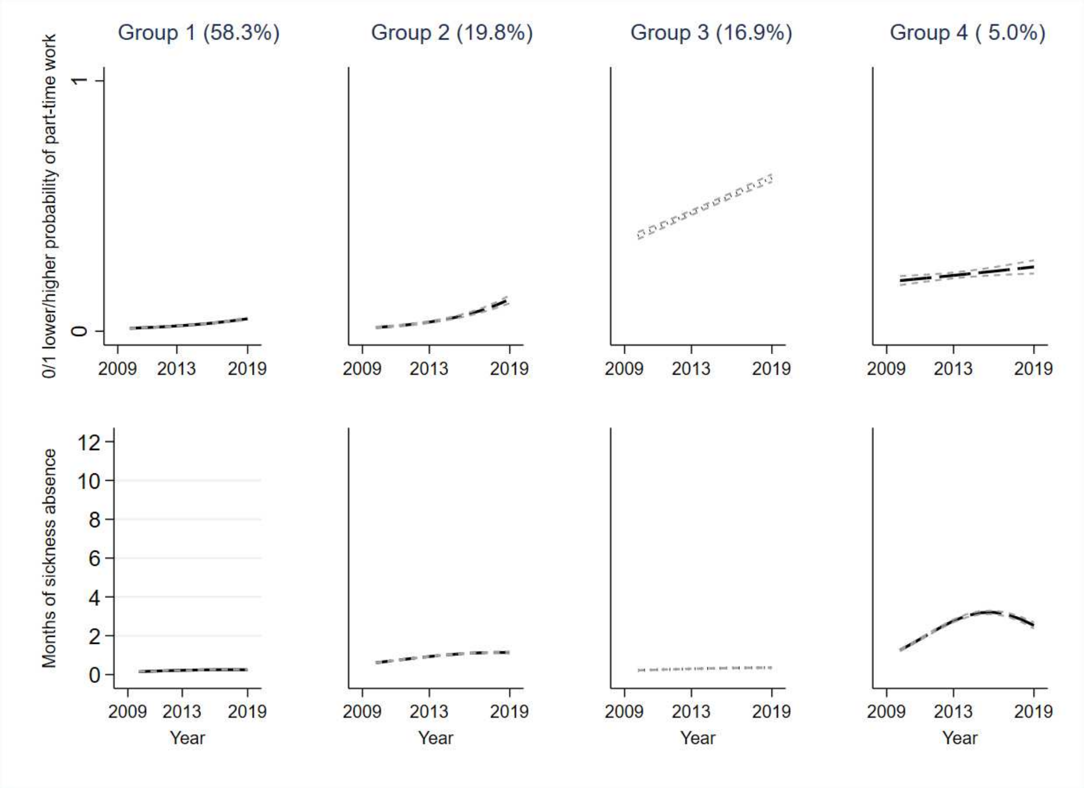

Supplemental Figure S1 Four clusters of trajectories of part-time work and months of sickness absence (95% CI are shown as dotted lines, but poorly visible due to being very narrow)

BMJ Publishing Group Limited (BMJ) disclaims all liability and responsibility arising from any reliance placed on this supplemental material which has been supplied by the author(s)

Supplemental material

Supplemental Table S2 Goodness of fit statistics of group-based trajectory analysis models

	**Baseline age < 25 years (n = 1950)**					**Baseline age ≥25 and <40 years (n = 7437)**				
	**Smallest group**					**Smallest group**				
	**N**	**%**	**BIC**	**AIC**	**APP**	**N**	**%**	**BIC**	**AIC**	**APP**
2-cluster model	453	25	-13732.04	-13690.23	0.90	1790	25	-63056.56	-63004.71	0.93
3-cluster model	235	14	-13234.66	-13170.54	0.88	928	13	-59538.30	-59458.79	0.90
**4-cluster model***	95	6	-13196.39	-13109.97	0.78	**649**	**10**	**-58972.56**	**-58865.39**	**0.86**
**5-cluster model***	**80**	**5**	**-13183.34**	**-13074.62**	**0.83**	274	4	-58496.44	-58361.61	0.88
6-cluster model	70	4	-13142.51	-13011.84	0.81					
7-cluster model	17	1	-13118.99	-12965.66	0.93					
	**Baseline age ≥40 and <55 years (n = 10143)**					**Baseline age ≥55 years (n = 1430)**				
2-cluster model	2626	26	-94282.84	-94228.65	0.94	322	23	-12128.61	-12089.12	0.97
3-cluster model	1120	11	-89502.96	-89419.88	0.94	259	19	-11463.96	-11403.41	0.91
4-cluster model	597	6	-87992.44	-87880.46	0.91	154	10	-11295.51	-11213.90	0.91
**5-cluster model***	**535**	**5**	**-87053.32**	**-86912.44**	**0.91**	**139**	**10**	**-11202.11**	**-11099.44**	**0.84**
6-cluster model	277	3	-86516.63	-86346.85	0.87	54	4	-11094.27	-10970.53	0.89

* The models presented are shown in bold. BIC = Bayesian Information Criterion, AIC = Akaike Information Criterion, and APP = average posterior probability.

Supplemental material

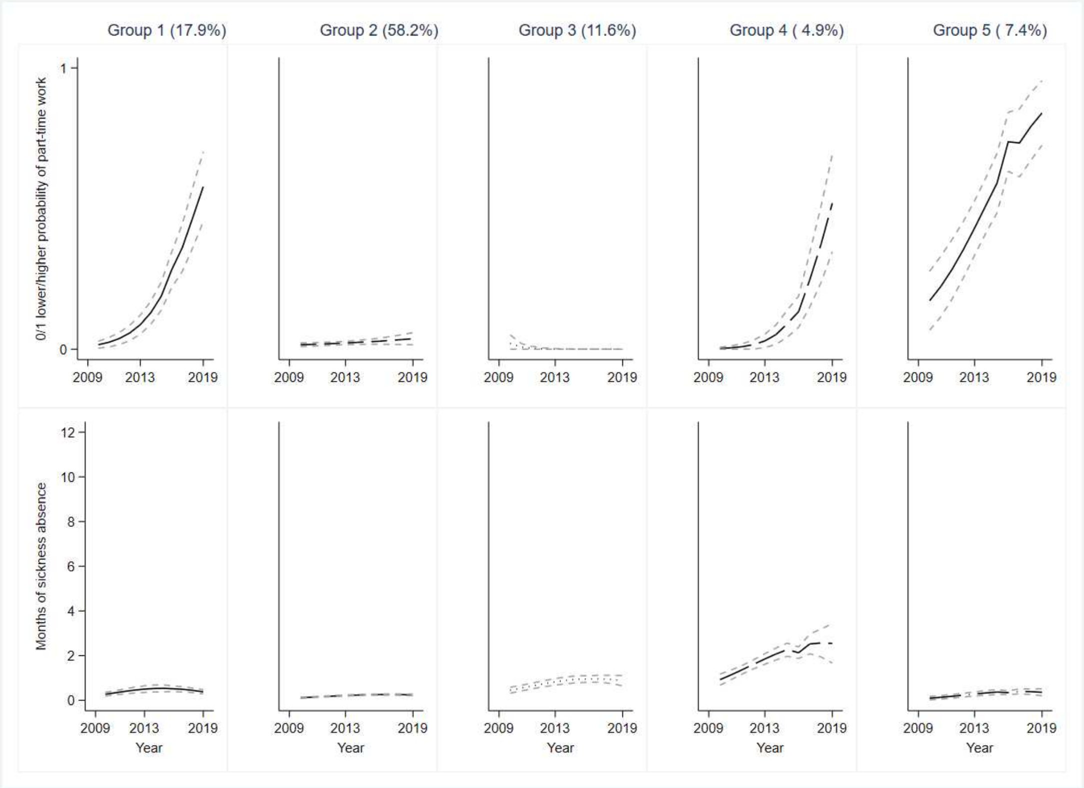

Supplemental Figure S2 Five clusters of trajectories of part-time work and months of sickness absence **among those with baseline age <25 years** (n = 1950) adjusted for time-variant night work (95% CI are shown as dotted lines, but poorly visible due to being very narrow)

Supplemental material

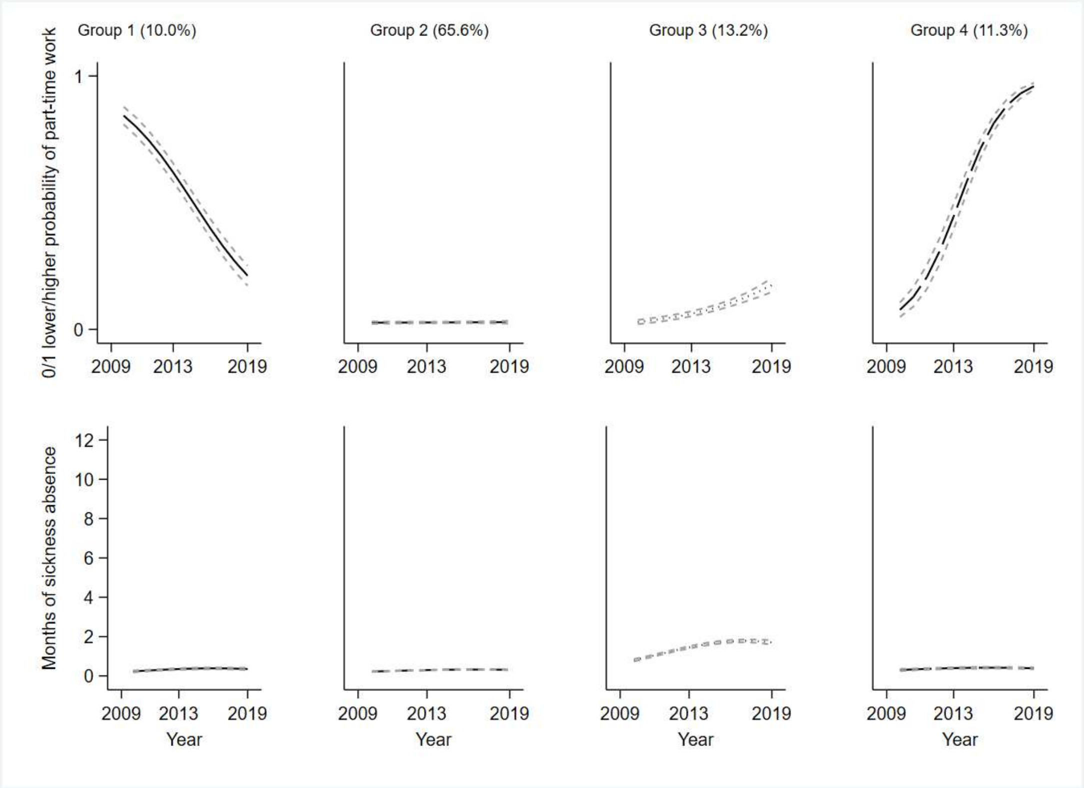

Supplemental Figure S3 Four clusters of trajectories of part-time work and months of sickness absence **among those with baseline age ≥25 and <40 years** (n = 7437) adjusted for time-variant night work (95% CI are shown as dotted lines, but poorly visible due to being very narrow)

Supplemental material

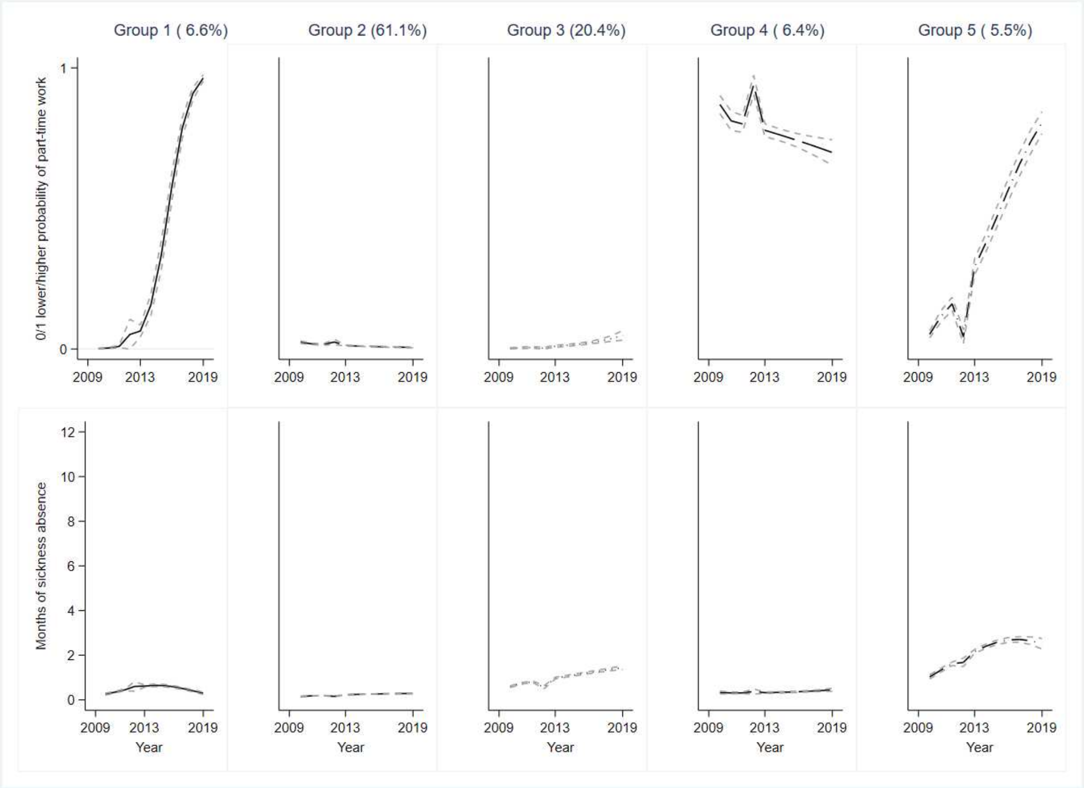

Supplemental Figure S4 Five clusters of trajectories of part-time work and months of sickness absence **among those with baseline age ≥40 and <55 years** (n = 10143) adjusted for time-variant night work (95% CI are shown as dotted lines, but poorly visible due to being very narrow)

Supplemental material

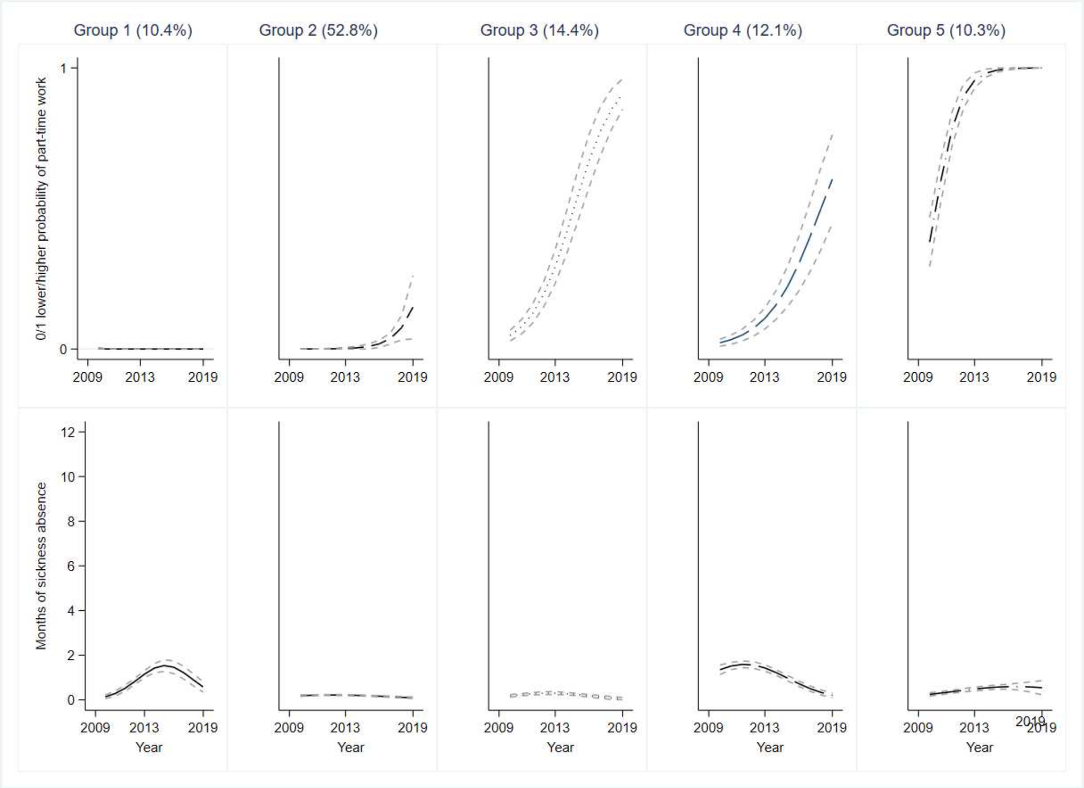

Supplemental Figure S5 Five clusters of trajectories of part-time work and months of sickness absence **among those with baseline age ≥55 years** (n = 1430) adjusted for time-variant night work (95% CI are shown as dotted lines, but poorly visible due to being very narrow)
