# Supplementary material for: Concurrent trajectories of part-time work and sickness absence: a longitudinal cohort study over 11 years among shift working hospital employees
Source: BMJ Open. 2023 Sep 25;13(9):e072987. doi: 10.1136/bmjopen-2023-072987 (PMC10533668; doi:10.1136/bmjopen-2023-072987)
Supplement: Author's manuscript [file bmjopen-2023-072987.draft_revisions.pdf]

BMJ Open

BMJ Open is committed to open peer review. As part of this commitment we make the peer review history of every article we publish publicly available.

When an article is published we post the peer reviewers' comments and the authors' responses online. We also post the versions of the paper that were used during peer review. These are the versions that the peer review comments apply to.

The versions of the paper that follow are the versions that were submitted during the peer review process. They are not the versions of record or the final published versions. They should not be cited or distributed as the published version of this manuscript.

BMJ Open is an open access journal and the full, final, typeset and author-corrected version of record of the manuscript is available on our site with no access controls, subscription charges or pay-per-view fees (http://bmjopen.bmj.com).

If you have any questions on BMJ Open's open peer review process please email info.bmjopen@bmj.com

BMJ Open

Concurrent trajectories of part-time work and full-time sickness absence among a cohort of shift working hospital employees

Journal:	*BMJ Open*
Manuscript ID	bmjopen-2023-072987
Article Type:	Original research
Date Submitted by the Author:	20-Feb-2023

Complete List of Authors:	Ropponen, Annina; Finnish Institute of Occupational Health; Karolinska Institute, Department of Clinical Neuroscience<br>Ervasti, Jenni; Finnish Institute of Occupational Health, Topeliuksenkatu 41 a A, FI-00250, Helsinki, Finland<br>Harma, Mikko; Finnish Institute of Occupational Health, Work Ability and Work Careers

Keywords:	EPIDEMIOLOGY, Human resource management < HEALTH SERVICES ADMINISTRATION & MANAGEMENT, OCCUPATIONAL & INDUSTRIAL MEDICINE

SCHOLARONE™
Manuscripts

I, the Submitting Author has the right to grant and does grant on behalf of all authors of the Work (as defined in the below author licence), an exclusive licence and/or a non-exclusive licence for contributions from authors who are: i) UK Crown employees; ii) where BMJ has agreed a CC-BY licence shall apply, and/or iii) in accordance with the terms applicable for US Federal Government officers or employees acting as part of their official duties; on a worldwide, perpetual, irrevocable, royalty-free basis to BMJ Publishing Group Ltd ("BMJ") its licensees and where the relevant Journal is co-owned by BMJ to the co-owners of the Journal, to publish the Work in this journal and any other BMJ products and to exploit all rights, as set out in our licence.

The Submitting Author accepts and understands that any supply made under these terms is made by BMJ to the Submitting Author unless you are acting as an employee on behalf of your employer or a postgraduate student of an affiliated institution which is paying any applicable article publishing charge ("APC") for Open Access articles. Where the Submitting Author wishes to make the Work available on an Open Access basis (and intends to pay the relevant APC), the terms of reuse of such Open Access shall be governed by a Creative Commons licence – details of these licences and which Creative Commons licence will apply to this Work are set out in our licence referred to above.

Other than as permitted in any relevant BMJ Author's Self Archiving Policies, I confirm this Work has not been accepted for publication elsewhere, is not being considered for publication elsewhere and does not duplicate material already published. I confirm all authors consent to publication of this Work and authorise the granting of this licence.

For peer review only

Concurrent trajectories of part-time work and full-time sickness absence among a cohort of shift working hospital employees

Authors: Annina Ropponen[1,2], Jenni Ervasti[1], Mikko Härmä[1]

[1] Finnish Institute of Occupational Health, Helsinki, Finland

[2] Division of Insurance Medicine, Department of Clinical Neuroscience, Karolinska Institute, Stockholm, Sweden

Corresponding author: Annina Ropponen, Finnish Institute of Occupational Health, P.O. Box 18, 00032 Finnish Institute of Occupational Health, Finland, email. annina.ropponen@ttl.fi

Word count: 2575.

For peer review only

Abstract

Objectives: To investigate the concurrent changes in part-time work and sickness absence (SA) in health care. Another aim was to investigate the role of age and sex on different concurrent trajectory groups.

Design: Prospective cohort study.

Setting: Public hospital districs (n = 10) and cities (n = 11) in Finland.

Participants: Payroll-based objective working hour data of health care sector in Finland for 28969 employees in 2008-2019 were utilized. Lack of baseline SA (≥14 days), three consecutive years of data and working shifts were set for final sample.

Primary outcomes: Part-time work (yes/no) and months of SA.

Measures: Group-based trajectory modelling to identify concurrent changes in part-time work, and months of SA while controlling time-variant amount of night work and multinomial regression models for relative risk (RR) with 95% confidence intervals (CI) were used.

Results: Four-group trajectory model was the best solution: Group 1 (61.2%) with full-time work and no SA, Group 2 (16.9%) with slowly increasing probability of part-time work and low but mildly increasing SA, Group 3 (17.6%) with increasing part-time work and no SA, and Group 4 (4.3%) with fluctuating, increasing part-time work and highest and increasing levels of SA. Men had a lower (RR 0.49-0.75), and older age groups had a higher likelihood (RRs 1.32-3.79) of belonging to trajectory groups 2-4.

Conclusions Most of the sample were in the trajectory group having full-time work and no SA. Probability of part-time work increased over time being linked with concurrent low increase or no SA. A minor group of employees had both an increased probability of part-time work and SA. Other solutions than part-time work might merit attention to promote sustainable working life among health care employees.

For peer review only

Article summary

Strengths and limitations of this study

- Payroll-based register data of working hours enabled objective assessment of part-time work and sickness absence.
- Homogenous sample of hospital employees working in specialized healthcare services or at primary health care, including e.g., health centers, and local hospitals for elderly and chronic ill people providing 24/7 care, or home care for disabled.
- Group-based trajectory model (GBTM) to identify concurrent trajectories of part-time work and mean number of SA months/years for the follow-up from 2009 to 2019 to estimate a distinct pattern over time.
- Lack of information on the reasons for part-time work and no diagnosis for sickness absence.

Keywords: shift work, part-time work, hospital, sick leave, longitudinal study

For peer review only

Background

Health care sector is facing major challenges due to population ageing, increased need for care, and economic challenges combined with staff shortage. The ageing population affects both ensuring sufficient levels of nursing staff, but also on growing demands for care for the ageing population [1 2]. The existing need for longer work careers [3] combined with increasing turnover rates emphasizes the urgent need to understand working hours in association with work capacity and sustainable working life. This need for understanding for associations between working hours and work capacity builds on the Eurofound's definition of sustainable work which calls for "working and living conditions that are such that they support people in engaging and remaining in work throughout an extended working life" [4]. Part-time work, or a change from night work to day-time work, may provide a possibility to decrease workload due to demanding working conditions [5 6] or to arrange an more time for care for small children or other dependents. Reasons of switching to part-time work may also be health-related, i.e., being based on part-time sickness absence (SA) or disability pension (DP) [7 8]. However, studies on the role of part-time work in relation future long-time trajectories of SA are lacking.

Working part-time may, besides influencing the income, have long-term consequences on career and pension [9]. On the other hand, shorter working hours and increased time for recovery could lengthen the work careers [10]. Due to an emergent need to increase employment rates and promote sustainable working life among the working population [11 12], longitudinal studies investigating the role of part-time work are important. Many studies have reported associations between individual sociodemographic, socioeconomic, health- and work-related factors including part-time work for labor market exit due to SA/DP [13 14]. However, until now, studies with longitudinal designs to investigate associations between part-time work and sustainable working life have mainly been based on survey data [12 15-20].

For peer review only

A hypothesis of this study was that due to ageing, older employees may choose part-time work due to decreased health (reflected in higher sickness absence) more often than younger employees. We also hypothesize that part-time work without increased sickness absence might be more frequent among younger employees than older ones. The possibility for part-time work, that is, to shorten working hours when having health problems and while compensated by social benefits, may extend and/or maintain the working lives of the older employees. However, part-time work and full-time SA may also both increase along age. Another aspect related to age is night work, i.e., older employees may wish to work more during the days than nights [21]. Also age-related trends in the associations of night work and health have been identified [22 23]. Therefore, based on the assumption that younger and older employees might react differently to night work [24 25], one should control night work in the when investigating age-related differences in part-time work and SA. This is relevant, especially in longitudinal settings in which working during the nights may decrease due to possibilities to select more favourable working hours [26 27].

This study aimed to investigate the concurrent changes in part-time work and SA among employees in health care without SA (≥14 subsequent days) at the baseline while controlling the amount of night work. Another aim was to investigate the role of age and sex on different concurrent trajectory groups.

Methods

This study was based on the ongoing Working Hours in the Finnish Public Sector study (WHFPS) [28 29] with payroll-based register data of working hours for 2008-2019. For this study, we restricted the sample to those with ≥31 work shifts/year in three consecutive years during 2008-2019. Then the sample was restricted to those without sickness absence (SA, ≥14 consequent days) at baseline in 2008. Last, only those with shift work contract were included (Figure 1). The final sample was 28969 shift working employees in 10 hospital districts and 11 cities using Titania® shift scheduling software. In Finland, the hospital districts (45% of the final sample) are responsible for specialized healthcare

services providing both specialized outpatient- and inpatient care. The health care employees in the cities (55% of the final sample) are those working at primary health care, including e.g., health centers, and local hospitals for elderly and chronic ill people providing 24/7 care, or home care for disabled [29].

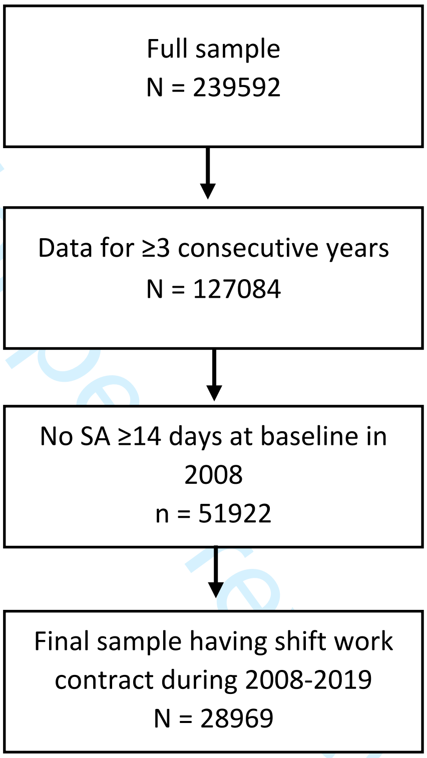

Figure 1 Sample selection

In this study, we utilized annual working hour and SA data from 2008-2019 as has been described in detail before [28 29]. Part-time work was based on work contract information and coded yes/no for each year based on annual median. Then annual SA of any length were measured in months/year for follow-up from 2009 to 2019. For the baseline 2008, the short SAs (≤3 days) were estimated. We also utilized the proportion of night work of all work shifts/year to estimate the time-varying effect of night work as a proxy of shift work in this study. Baseline age (in 2008 and years categorized into < 25 years of age, ≥25 and <40 years, ≥40 and <55 years, and >55 years based on distribution), and sex were used in the analyses.

For peer review only

Statistical analyses

First, we calculated the descriptive characteristics for the study sample. Then using a group-based trajectory model (GBTM), we identified concurrent trajectories of part-time work and mean number of SA months/years for the follow-up from 2009 to 2019 while accounting night work as time varying covariate [30]. The GBTM method enables to identify groups of individuals (trajectory groups) which follow a distinct pattern over time. The linear polynomial model was applied. Bayesian information criterion (BIC), Akaike Information Criteria (AIC) and average posterior probability (APP) were used to determine the best-fitting model. Third, we ran multinomial regression models for relative risk (RR) and 95% Confidence Intervals (CI) using sex and age groups for predicting the group membership for each trajectory group. All statistical analyses were conducted with Stata 17.1 MP.

Results

Among the final sample of 28969 shift working employees, mean age at baseline in 2008 was 39.8 years (SD 10.7), and 92% were women. In average, 7% worked part-time and even there were no ≥14 days SA at baseline in 2008, 34% had had at least one short SA (≤3 days) during the baseline. The amount of night shifts was 8% of all shifts. During the follow-up (from 2009 to 2019), the mean number of SA months varied from 0.3 months in 2009 to 0.6 months in 2018 (range 0-12 months).

A four-cluster model was chosen based on BIC/AIC values and parsimony for concurrent changes in part-time work and months of SA (Table 1).

Table 1 Goodness of fit statistics of group-based trajectory analysis models

	Smallest group				
	N	%	BIC	AIC	APP
2-cluster model	4204	16.5	-190414.9	-190352.8	0.90
3-cluster model	3795	14.4	-181308.3	-181213.1	0.89
**4-cluster model***	**1099**	**4.3**	**-179517.1**	**-179388.9**	**0.87**
5-cluster model	1156	4.4	-175817.0	-175655.6	0.86
6-cluster model	1134	4.4	-175451.2	-175256.7	0.86
7-cluster model	688	3.7	-17117.5	-173890.0	0.79

* The models presented are shown in bold. BIC = Bayesian Information Criterion, AIC = Akaike Information Criterion, and APP = average posterior probability.

The concurrent trajectories were (Figure 1):

For peer review only

1. Group 1 (61.2%) with full-time work and no sickness absence.
2. Group 2 (16.9) with slowly increasing probability of part-time work and low but mildly increasing sickness absence.
3. Group 3 (17.6%) with increasing part-time work and no sickness absence.
4. Group 4 (4.3%) with fluctuating but increasing part-time work and highest and increasing levels of sickness absence.

The regression models (Table 2) showed that compared to the youngest age group (<25 years of age at the baseline), the older age groups had increased likelihood of belonging to the trajectory groups 2-4 (in comparison to trajectory group 1). Men had lower likelihood of belonging to trajectory groups 2-4 than women (Table 2).

Table 2 Sex and age group associated with cluster membership (part-time work and months of sickness absence). Multinomial regression (ref=Cluster 1 'Full-time work and no sickness absence', n=2124). Relative risk ratio (RR) with 95% confidence intervals (CI)

	**Group 2** Slowly increasing probability of part-time work and low but mildly increasing sickness absence		**Group 3** Increasing part-time work and no sickness absence		**Group 4** fluctuating but increasing part-time work and highest and increasing levels of sickness absence	
	**RR***	**95%CI**	**RR**	**95%CI**	**RR**	**95%CI**
Women	1		1		1	
Men	**0.74**	**0.63, 0.86**	**0.75**	**0.65, 0.87**	**0.49**	**0.34, 0.71**
Age <25 years	1		1		1	
25-39 years	**1.32**	**1.13, 1.54**	**1.56**	**1.36, 1.80**	**2.36**	**1.56, 3.56**
40-54 years	**1.83**	**1.58, 2.12**	1.10	0.95, 1.26	**3.79**	**2.54, 5.66**
≥55 years	**1.69**	**1.39, 2.07**	**1.53**	**1.27, 1.85**	**2.99**	**1.83, 4.88**

* Statistically significant RR with 95%CI in boldface

In the supplemental material, we present the concurrent trajectories without adjusting for time-variant night work (Supplemental Figure 1), and for each age group separately (Supplemental Figures S2-5) and their fit indexes in the supplemental tables S1-2. Although trajectory groups 1-3 are similar than adjusting for time-variant night work, the group 4 indicates some changes in the probability of

For peer review only

part-time work. Except for those aged 25-39 years with four trajectory groups, all other age groups were best fitted to a five-group trajectory solution. The concurrent changes of part-time work and SA varied between age groups: among the oldest age group, ≥55 years, three out of five trajectory groups had increase in part-time work associated with low or no SA. The concurrent trajectories with decrease in part-time work (transition to full-time) with no SA were identified among middle age groups (25-54 years).

Discussion

This longitudinal study of almost 29 000 Finnish employees in health care sector without long-term (≥14 subsequent days) SA at baseline, aimed to investigate the concurrent changes in part-time work and SA across 11 years (from 2009 to 2019). We controlled for the amount of night work during the follow-up as a proxy for shift work. After identifying the trajectory clusters, we investigated the role of age and sex on the identified concurrent trajectories. Four trajectory groups were detected: Group 1 (61.2%) with full-time work and no SA, Group 2 (16.9) with slowly increasing probability of part-time work and low but mildly increasing SA, Group 3 (17.6%) with increasing part-time work and no SA, and Group 4 (4.3%) with fluctuating but increasing part-time work and highest and increasing levels of SA. Men had a lower likelihood and older age groups had a higher likelihood of belonging to trajectory groups 2-4 in comparison to group 1. This is among the first studies to investigate the concurrent changes of part-time work and SA across 11 years of follow-up while controlling for night work among health care employees. Hence, this study adds to the earlier knowledge based on survey data [12 15-20].

The largest trajectory group (61%) worked full-time and had no to minimal amount of SA reflecting the well-known trend of women working mostly full-time in the Nordic countries [9]. However, we also detected two moderate size groups (Trajectory Groups 2 and 3) with increasing probability of part-time work and low but mildly increasing or no SA. These might be indicative for our hypotheses that a) along ageing, older employees may choose part-time work due to decreasing health (with higher

SA), or b) part-time work with low/no SA might appear among younger employees due to needs to balance work and life. Since the fourth trajectory group, although small (4%), indicated increase both in the probability of part-time work and SA, it seems to support the above-mentioned hypothesis, and the assumption that both part-time work and full-time SA increase along age. Our Supplemental figure S1 indicated that overall, four group trajectory solution was the best even without time variant night work, but the probability of part-time work was less likely indicating that transition to day work may be another strategy to affect the working hours along age [21]. This might be an important notion for workplaces and occupational health care since age-related trends in the associations of night work and health exist [22 23] and perhaps modifications to working hours could promote sustainable working life [11 12] if part-time work would be compensated with social benefits.

We were specifically interested on the role of sex and age on these concurrent changes of part-time work and SA. Men had a lower likelihood of belonging to trajectory groups 2-4 characterized by increases in part-time work and with or without SA. This is in line with earlier findings indicating that e.g., a typical employee having long-term SA is a woman at her 50's and working in the healthcare sector [31 32]. This might be because women have higher SA rates than men [33-35], but also because women outnumber men in the healthcare sector (in our study, only 8% were men). Another influential, well-known fact is that men and women work part-time due to different reasons such as child care or health [9] and even societal factors such as availability of daycare for children or salary levels can play a role [36-38]. Older age groups had a higher likelihood of belonging to trajectory groups 2-4 compared to group 1. There are some possible explanations. First, the concurrent increase of part-time work and full-time SA along age. Alternatively age-related trends of reacting to night work [24 25], i.e., across age groups the concurrent changes of part-time work and SA were different as shown in our supplement figures.

We had access to objective working hour data including SA from 2008 to 2019 in total almost 240 000 Finnish employees in health care sector. This unique dataset enabled us to restrict the

For peer review only

sample to those working shift work, having complete data for ≤3 consecutive years during 2009-2019, and without baseline ≥14 days SA. These are evident strengths although the final sample size was around 29 000 employees. Such data is free from reporting or memory bias adding to earlier knowledge based on mainly survey data [12 15-20]. However, a limitation is that we had no information on the reasons for part-time work which may be due to e.g., health, personal matters, or unavailability of full-time work [39 40]. Furthermore, we only had information of SA without diagnosis and no information on part-time sickness absence. Since part-time work may relate to health status, i.e., being based on part-time SA or DP [7 8], these aspects should be accounted in further studies. Another limitation might be generalizability of these findings. In Finland, and in Nordic countries, work at healthcare is organized mostly via irregular working hours, i.e., shift work with a non-standard working hour schedule with variation in start and finish times, lengths, and rest periods between work shifts [41]. On the other hand, SA are subsidized via welfare society model typical to Nordic countries. Hence our findings might be more generalizable to Nordic than other countries.

Conclusions

Working full-time and having no sickness absence were observed concurrently together. Probability of part-time work increased over time being linked with concurrent low increase or no sickness absence. A minor group of employees with both an increased probability of part-time work and sickness absence was also identified. Being men seemed to lower the likelihood of these probabilities whereas older ages increased. Other working hour solutions than part-time work might merit attention to promote sustainable working life among health care employees.

For peer review only

Statements and declarations

Acknowledgements

This work was supported by the Academy of Finland, DIGIHUM-programme grant number 329200.

Conflict of interest

The authors declare that they have no conflict of interest.

Author contributions

AR, MH, and JE contributed to the study's conception and design. Material preparation, data collection and analysis were performed by AR. The first draft of the manuscript was written by AR, and all the authors commented on previous versions of the manuscript. All the authors read and approved the final manuscript.

Availability of data and materials

The data that support the findings of this study are available from the Hospital districts and Cities but restrictions apply to the availability of these data, which were used under license for the current study, and so are not publicly available. Data are however available from the last author upon reasonable request and with permission of the Hospital districts and Cities.

Ethics approval

This study was fully based on employer-owned administrative employment register data that the Hospital Districts and Cities had permitted the access and applied pseudonymized identification numbers for research purposes. Using such data does not include any experimental protocols requiring approval. In Finland, research using such data does not need to undergo review by an ethics committee according to national legislation (Medical Research Act).

Consent to participate

For peer review only

The consent to participate was not applicable since the data comprised employer-owned employment information and was analyzed as administrative register data pseudonymized for research purposes.

Consent for publication

Not applicable.

For peer review only

Figure legend

Figure 1 Four clusters of trajectories of part-time work and months of sickness absence (SA) adjusted for time-variant night work (95% CI are shown as dotted lines, but poorly visible due to being very narrow)

For peer review only

References

1. Tursunbayeva A. Human resource technology disruptions and their implications for human resources management in healthcare organizations. *BMC Health Serv Res* 2019;19(1):268. doi: 10.1186/s12913-019-4068-3 [published Online First: 2019/05/01]
2. Smiley RA, Lauer P, Bienemy C, et al. The 2017 National Nursing Workforce Survey. *Journal of Nursing Regulation* 2018;9(3):S4-S88. doi: Doi 10.1016/S2155-8256(18)30131-5
3. Vanroelen C. Longer working careers and sustainable work: the issue of social inequality. *Society Health & Vulnerability* 2017;8(sup1):1332854. doi: Unsp 1332854

10.1080/20021518.2017.1332854

4. Eurofound. Sustainable working over the life course: concept paper. Publications Office of the European Union, Luxembourg, 2015.
5. Steinmetz S, de Vries DH, Tijdens KG. Should I stay or should I go? The impact of working time and wages on retention in the health workforce. *Hum Resour Health* 2014;12:23. doi: 10.1186/1478-4491-12-23 [published Online First: 2014/04/25]
6. Flodgren GM, Bidonde J. Effects and experiences of part-time work in the health- and community-care services: a scoping review. (Effekter og erfaringer av deltidsarbeid i helse- og omsorgstjenesten: en kartleggingsoversikt). Oslo: National Institute of Public Health, 2019.
7. Schram JL, Solovieva S, Leinonen T, et al. The influence of occupational class and physical workload on working life expectancy among older employees. *Scandinavian journal of work, environment & health* 2021;47(1):5-14. doi: 10.5271/sjweh.3919 [published Online First: 2020/09/01]
8. Ervasti J, Kausto J, Koskinen A, et al. Labor Market Participation Before and After Long-Term Part-Time Sickness Absence in Finland: A Population-Based Cohort Study. *Journal of Occupational and Environmental Medicine* 2020;62(4)
9. NIKK. Faktablad: Vem jobbar och när? Norway: Nordiska ministerrådet, 2018.
10. Härmä MI, Ilmarinen JE. Towards the 24-hour society--new approaches for aging shift workers? *Scand J Work Environ Health* 1999;25(6):610-5. doi: 10.5271/sjweh.488 [published Online First: 2000/07/07]
11. Health MoSAa. Eheä yhteiskunta ja kestävä hyvinvointi Sosiaali- ja terveysministeriön tulevaisuuskatsaus. Valtioneuvoston julkaisusarja 22/2018. Helsinki: Sosiaali- ja terveysministeriö, 2018.
12. de Wind A, van der Pas S, Blatter BM, et al. A life course perspective on working beyond retirement-results from a longitudinal study in the Netherlands. *BMC Public Health* 2016;16:499. doi: 10.1186/s12889-016-3174-y [published Online First: 2016/06/12]
13. Reinholdt S, Upmark M, Alexanderson K. Health-selection mechanisms in the pathway towards a disability pension. *Work* 2010;37(1):41-51. doi: 10.3233/WOR-2010-1055 [published Online First: 2010/09/23]
14. Ropponen A, Alexanderson K, Svedberg P. Part-time work or social benefits as predictors for disability pension: a prospective study of Swedish twins. *Int J Behav Med* 2014;21(2):329-36. doi: 10.1007/s12529-013-9303-4 [published Online First: 2013/03/13]
15. Lu W, Benson R, Glaser K, et al. Relationship between employment histories and frailty trajectories in later life: evidence from the English Longitudinal Study of Ageing. *J Epidemiol Community Health* 2017;71(5):439-45. doi: 10.1136/jech-2016-207887 [published Online First: 2016/12/04]
16. Sohier L, Van Ootegem L, Verhofstadt E. Well-Being During the Transition from Work to Retirement. *Journal of Happiness Studies* 2021;22(1):263-86. doi: 10.1007/s10902-020-00228-6
17. Komp-Leukkunen K. Life-histories in Finland: First results from the Survey of Health, Ageing and Retirement in Europe Working papers. Helsinki: Population Research Institute, 2021.

For peer review only

18. Ek E, Ala-Mursula L, Velázquez RG, et al. Employment trajectories until midlife associate with early social role investments and current work-related well-being. *Advances in Life Course Research* 2021;47:100391. doi: https://doi.org/10.1016/j.alcr.2020.100391
19. McDonough P, Worts D, Corna LM, et al. Later-life employment trajectories and health. *Advances in Life Course Research* 2017;34:22-33. doi: https://doi.org/10.1016/j.alcr.2017.09.002
20. de Wind A, Scharn M, Geuskens GA, et al. Predictors of working beyond retirement in older workers with and without a chronic disease - results from data linkage of Dutch questionnaire and registry data. *BMC Public Health* 2018;18(1):265. doi: 10.1186/s12889-018-5151-0 [published Online First: 2018/02/20]
21. Härmä M. Workhours in relation to work stress, recovery and health. *Scand J Work Environ Health* 2006;32(6):502-14.
22. Booker LA, Magee M, Rajaratnam SMW, et al. Individual vulnerability to insomnia, excessive sleepiness and shift work disorder amongst healthcare shift workers. A systematic review. *Sleep Med Rev* 2018;41:220-33. doi: 10.1016/j.smrv.2018.03.005 [published Online First: 2018/04/24]
23. Ritonja J, Aronson KJ, Matthews RW, et al. Working Time Society consensus statements: Individual differences in shift work tolerance and recommendations for research and practice. *Ind Health* 2019;57(2):201-12. doi: 10.2486/indhealth.SW-5 [published Online First: 2019/02/01]
24. Cai C, Vandermeer B, Khurana R, et al. The impact of occupational shift work and working hours during pregnancy on health outcomes: a systematic review and meta-analysis. *Am J Obstet Gynecol* 2019;221(6):563-76. doi: 10.1016/j.ajog.2019.06.051 [published Online First: 2019/07/06]
25. Stock D, Knight JA, Raboud J, et al. Rotating night shift work and menopausal age. *Hum Reprod* 2019;34(3):539-48. doi: 10.1093/humrep/dey390 [published Online First: 2019/02/13]
26. Hess M, Bauknecht J, Pink S. Working Hours Flexibility and Timing of Retirement: Findings from Europe. *J Aging Soc Policy* 2018;30(5):478-94. doi: 10.1080/08959420.2018.1500857 [published Online First: 2018/08/02]
27. Vanajan A, Bultmann U, Henkens K. Health-related Work Limitations Among Older Workers-the Role of Flexible Work Arrangements and Organizational Climate. *The Gerontologist* 2019 doi: 10.1093/geront/gnz073 [published Online First: 2019/06/01]
28. Harma M, Ropponen A, Hakola T, et al. Developing register-based measures for assessment of working time patterns for epidemiologic studies. *Scand J Work Environ Health* 2015;41(3):268-79. doi: 10.5271/sjweh.3492 [published Online First: 2015/03/20]
29. Härmä MI, Shiri R, Ervasti J, et al. National Recommendations for Shift Scheduling in Healthcare: A 5-Year Prospective Cohort Study on Working Hour Characteristics. 2022 doi: http://dx.doi.org/10.2139/ssrn.4084020
30. Nagin DS, Jones BL, Passos VL, et al. Group-based multi-trajectory modeling. *Stat Methods Med Res* 2018;27(7):2015-23. doi: 10.1177/0962280216673085 [published Online First: 2018/05/31]
31. Försäkrings A. Psykisk ohälsa i olika län, 2019.
32. samhällsmedicin Cfeo. Coronapandemin och psykisk hälsa. En prognos om kort- och långsiktiga effekter. Stockholm: Region Stockholm, 2020.
33. Borg K, Hensing G, Alexanderson K. Risk factors for disability pension over 11 years in a cohort of young persons initially sick-listed with low back, neck, or shoulder diagnoses. *Scand J Public Health* 2004;32(4):272-8. doi: 10.1080/14034940310019524
34. Haukenes I, Gjesdal S, Rortveit G, et al. Women's higher likelihood of disability pension: the role of health, family and work. A 5-7 years follow-up of the Hordaland Health Study. *BMC public health* 2012;12:720. doi: 10.1186/1471-2458-12-720

For peer review only

35. Mykletun A, Overland S, Dahl AA, et al. A population-based cohort study of the effect of common mental disorders on disability pension awards. *Am J Psychiatry* 2006;163(8):1412-8. doi: 10.1176/ajp.2006.163.8.1412 [published Online First: 2006/08/01]
36. Barbieri P, Cutuli G, Guetto R, et al. Part-time employment as a way to increase women's employment: (Where) does it work? *International Journal of Comparative Sociology* 2019;60(4):249-68. doi: 10.1177/0020715219849463
37. EuroStat. Being young in Europe today - labour market - access and participation 2020 [Available from: https://ec.europa.eu/eurostat/statistics-explained/index.php?title=Being_young_in_Europe_today_-_labour_market_-_access_and_participation.
38. Viikari-Juntura E, Leinonen T, Virta LJ, et al. Early part-time sick leave results in considerable savings in social security costs at national level: an analysis based on a quasi-experiment in Finland. *Scand J Work Environ Health* 2019;45(2):203-08. doi: 10.5271/sjweh.3780 [published Online First: 2018/10/20]
39. Pech C, Klainot-Hess E, Norris D. Part-time by Gender, Not Choice: The Gender Gap in Involuntary Part-time Work. *Sociological Perspectives* 2021;64(2):280-300. doi: 10.1177/0731121420937746
40. Rui F, Jennifer G, Peter CC. *Research Square* 2021 doi: 10.21203/rs.3.rs-390046/v1
41. Sallinen M, Kecklund G. Shift work, sleep, and sleepiness - differences between shift schedules and systems. *Scand J Work Environ Health* 2010;36(2):121-33. doi: 10.5271/sjweh.2900 [published Online First: 2010/02/02]

For peer review only

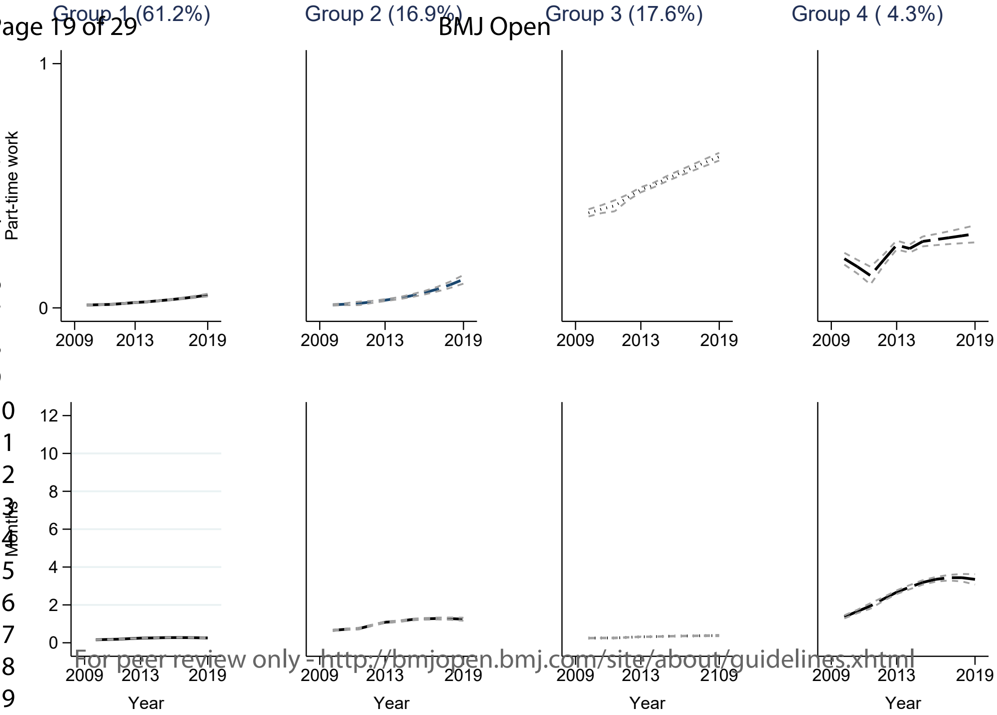
Group 1 (61.2%)
Group 2 (16.9%)
Group 3 (17.6%)
Group 4 (4.3%)
Part-time work
1
0
2009
2013
2019
Months
12
10
8
6
4
2
0
2009
2013
2019
2109
Year

Supplemental material

Concurrent trajectories of part-time work and full-time sickness absence among a cohort of shift working hospital employees

For peer review only

Supplemental material

Supplemental Table S1 Goodness of fit statistics of group-based trajectory analysis models

	Smallest group				
	N	%	BIC	AIC	APP
2-cluster model	5461	17	-263123.4	-263077.8	0.92
3-cluster model	5184	16	-254453.0	-254382.6	0.91
**4-cluster model***	**1671**	**5**	**-250141.8**	**-250046.5**	**0.91**
5-cluster model	919	4	-248427.7	-248307.6	0.84
6-cluster model	920	4	-244756.5	-244611.4	0.83

* The models presented are shown in bold. BIC = Bayesian Information Criterion, AIC = Akaike Information Criterion, and APP = average posterior probability.

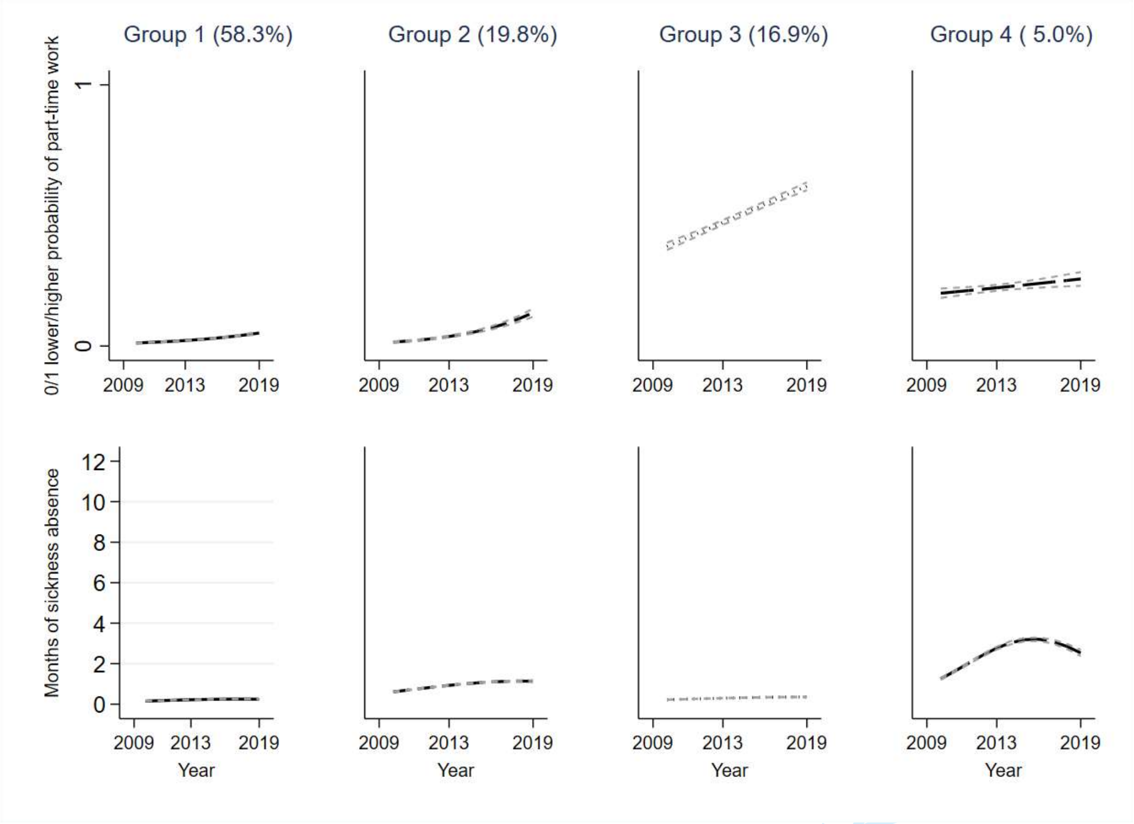

Supplemental Figure S1 Four clusters of trajectories of part-time work and months of sickness absence (SA) (95% CI are shown as dotted lines, but poorly visible due to being very narrow)

Supplemental material

Supplemental Table S2 Goodness of fit statistics of group-based trajectory analysis models

	**Baseline age < 25 years (n = 1950)**					**Baseline age ≥25 and <40 years (n = 7437)**				
	**Smallest group**					**Smallest group**				
	**N**	**%**	**BIC**	**AIC**	**APP**	**N**	**%**	**BIC**	**AIC**	**APP**
2-cluster model	453	25	-13732.04	-13690.23	0.90	1790	25	-63056.56	-63004.71	0.93
3-cluster model	235	14	-13234.66	-13170.54	0.88	928	13	-59538.30	-59458.79	0.90
**4-cluster model***	95	6	-13196.39	-13109.97	0.78	**649**	**10**	**-58972.56**	**-58865.39**	**0.86**
**5-cluster model***	**80**	**5**	**-13183.34**	**-13074.62**	**0.83**	274	4	-58496.44	-58361.61	0.88
6-cluster model	70	4	-13142.51	-13011.84	0.81					
7-cluster model	17	1	-13118.99	-12965.66	0.93					
	**Baseline age ≥40 and <55 years (n = 10143)**					**Baseline age ≥55 years (n = 1430)**				
2-cluster model	2626	26	-94282.84	-94228.65	0.94	322	23	-12128.61	-12089.12	0.97
3-cluster model	1120	11	-89502.96	-89419.88	0.94	259	19	-11463.96	-11403.41	0.91
4-cluster model	597	6	-87992.44	-87880.46	0.91	154	10	-11295.51	-11213.90	0.91
**5-cluster model***	**535**	**5**	**-87053.32**	**-86912.44**	**0.91**	**139**	**10**	**-11202.11**	**-11099.44**	**0.84**
6-cluster model	277	3	-86516.63	-86346.85	0.87	54	4	-11094.27	-10970.53	0.89

* The models presented are shown in bold. BIC = Bayesian Information Criterion, AIC = Akaike Information Criterion, and APP = average posterior probability.

For peer review only

For peer review only - http://bmjopen.bmj.com/site/about/guidelines.xhtml

Supplemental material

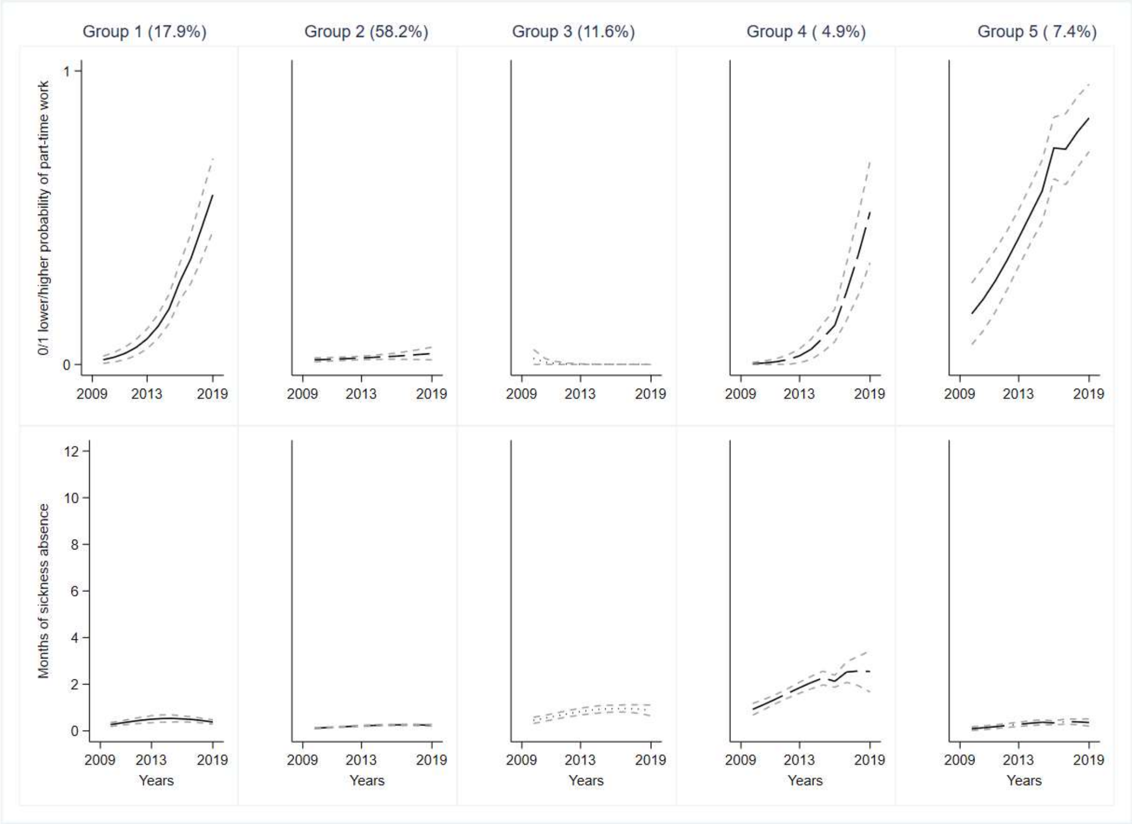

Supplemental Figure S2 Five clusters of trajectories of part-time work and months of sickness absence **among those with baseline age <25 years** (n = 1950) adjusted for time-variant night work (95% CI are shown as dotted lines, but poorly visible due to being very narrow)

Supplemental material

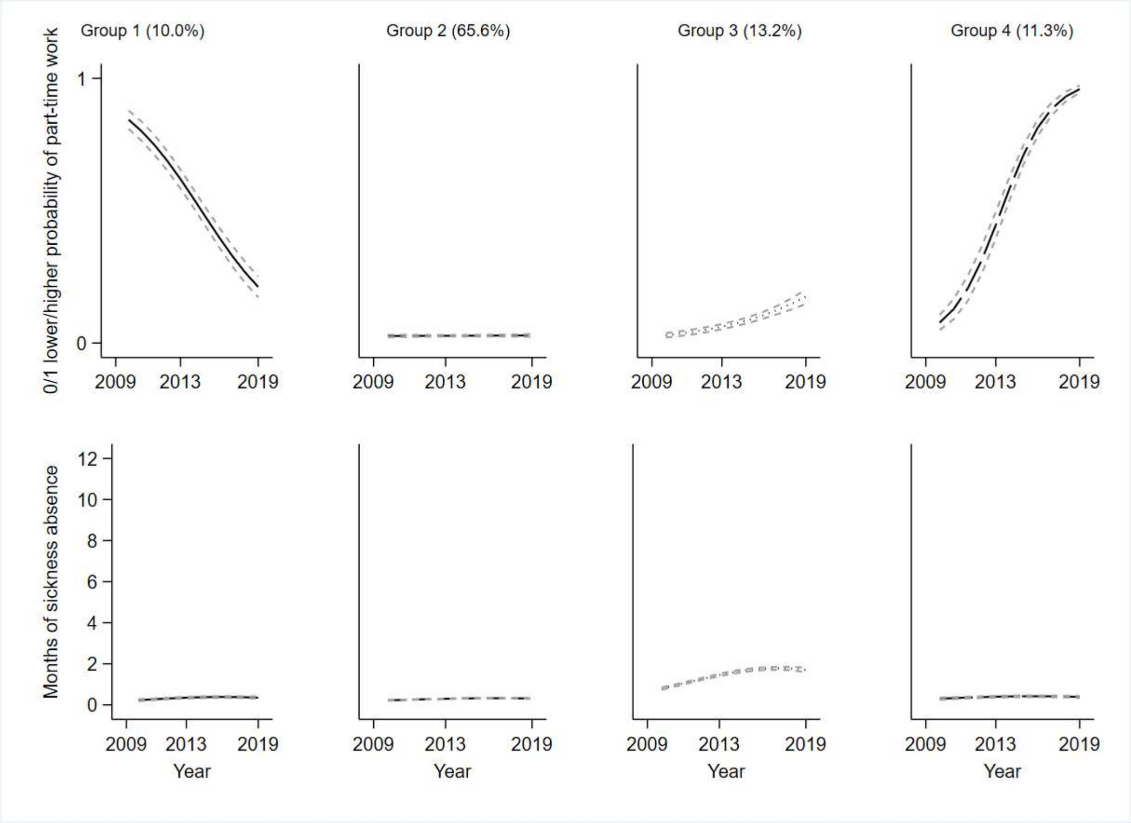

Supplemental Figure S3 Four clusters of trajectories of part-time work and months of sickness absence **among those with baseline age ≥25 and <40 years** (n = 7437) adjusted for time-variant night work (95% CI are shown as dotted lines, but poorly visible due to being very narrow)

Supplemental material

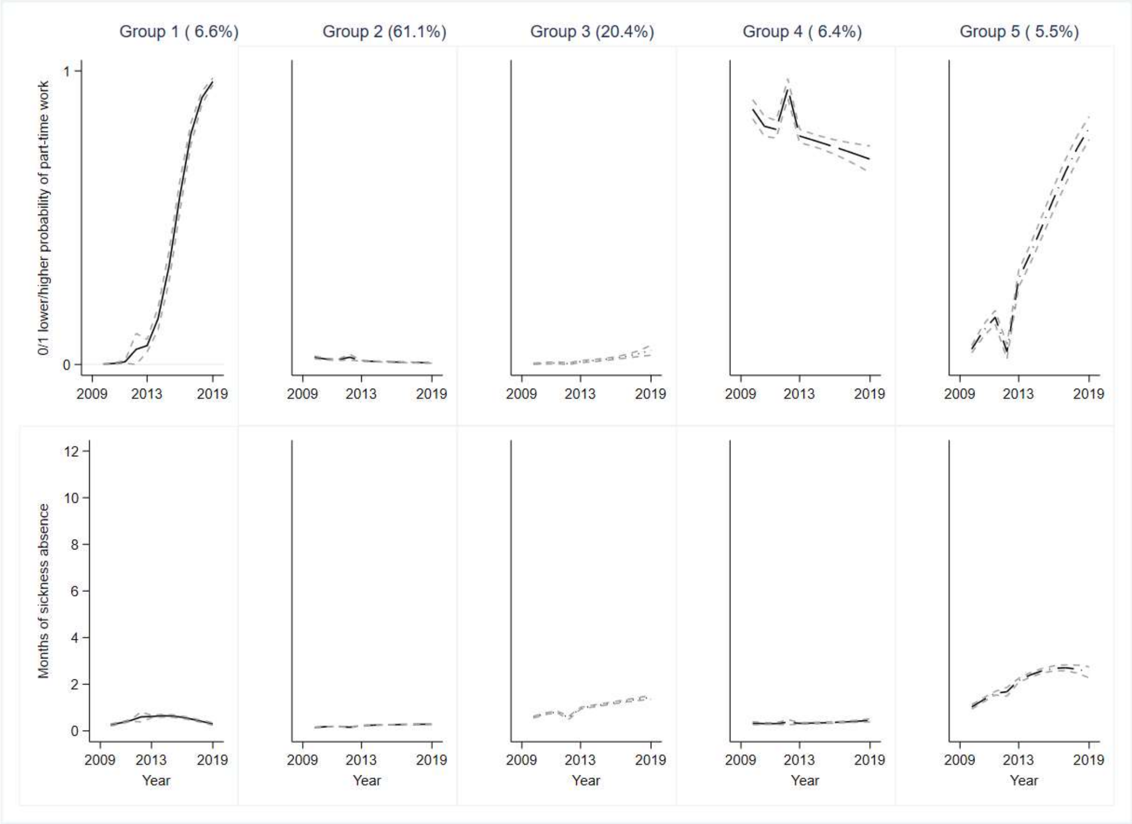

Supplemental Figure S4 Five clusters of trajectories of part-time work and months of sickness absence **among those with baseline age ≥40 and <55 years** (n = 10143) adjusted for time-variant night work (95% CI are shown as dotted lines, but poorly visible due to being very narrow)

Supplemental material

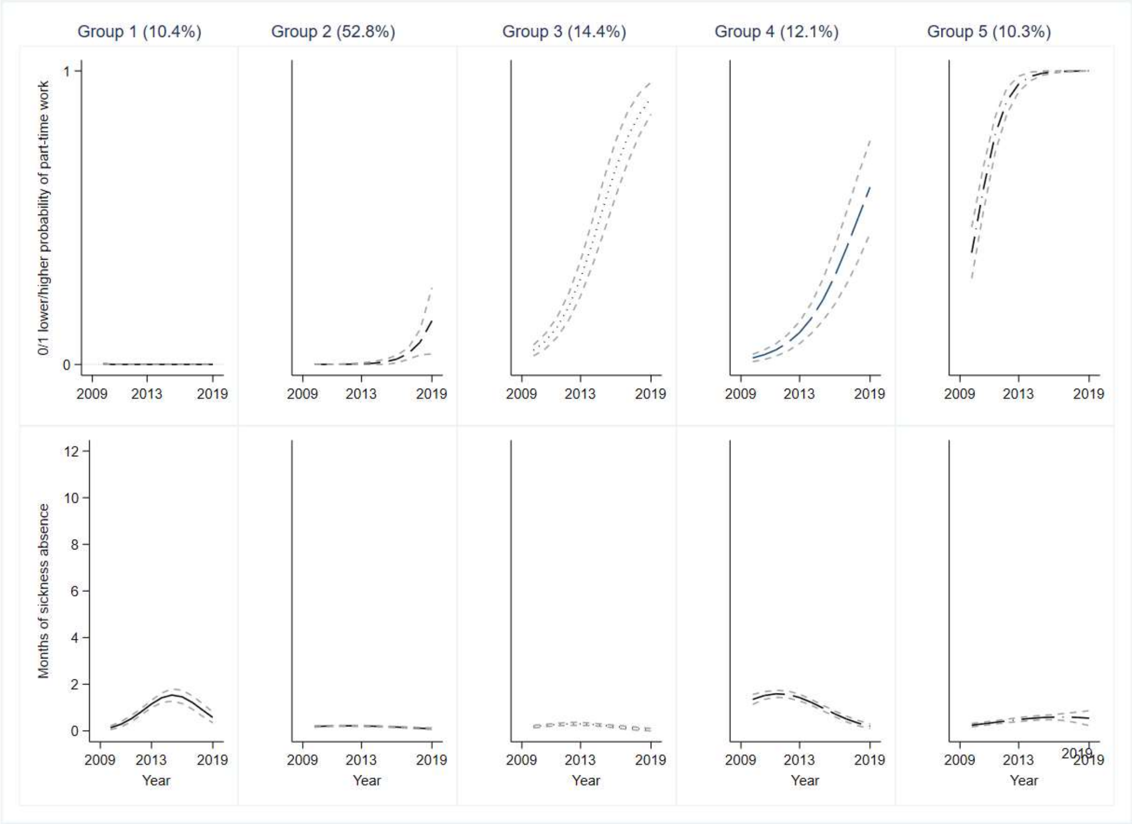

Supplemental Figure S5 Five clusters of trajectories of part-time work and months of sickness absence **among those with baseline age ≥55 years** (n = 1430) adjusted for time-variant night work (95% CI are shown as dotted lines, but poorly visible due to being very narrow)

Reporting checklist for cohort study.

Based on the STROBE cohort guidelines.

Instructions to authors

Complete this checklist by entering the page numbers from your manuscript where readers will find each of the items listed below.

Your article may not currently address all the items on the checklist. Please modify your text to include the missing information. If you are certain that an item does not apply, please write "n/a" and provide a short explanation.

Upload your completed checklist as an extra file when you submit to a journal.

In your methods section, say that you used the STROBE cohortreporting guidelines, and cite them as:

von Elm E, Altman DG, Egger M, Pocock SJ, Gotzsche PC, Vandenbroucke JP. The Strengthening the Reporting of Observational Studies in Epidemiology (STROBE) Statement: guidelines for reporting observational studies.

For peer review only

		Reporting Item	Page Number
**Title and abstract**			
Title	#1a	Indicate the study’s design with a commonly used term in the title or the abstract	1
Abstract	#1b	Provide in the abstract an informative and balanced summary of what was done and what was found	2
**Introduction**			
Background / rationale	#2	Explain the scientific background and rationale for the investigation being reported	4
Objectives	#3	State specific objectives, including any prespecified hypotheses	5
**Methods**			
Study design	#4	Present key elements of study design early in the paper	5
Setting	#5	Describe the setting, locations, and relevant dates, including periods	5

		of recruitment, exposure, follow-up, and data collection	
Eligibility criteria	#6a	Give the eligibility criteria, and the sources and methods of selection of participants. Describe methods of follow-up.	5
Eligibility criteria	#6b	For matched studies, give matching criteria and number of exposed and unexposed	6
Variables	#7	Clearly define all outcomes, exposures, predictors, potential confounders, and effect modifiers. Give diagnostic criteria, if applicable	7
Data sources / measurement	#8	For each variable of interest give sources of data and details of methods of assessment (measurement). Describe comparability of assessment methods if there is more than one group. Give information separately for for exposed and unexposed groups if applicable.	6
Bias	#9	Describe any efforts to address potential sources of bias	10
Study size	#10	Explain how the study size was arrived at	5
Quantitative variables	#11	Explain how quantitative variables were handled in the analyses. If applicable, describe which groupings were chosen, and why	6
Statistical methods	#12a	Describe all statistical methods, including those used to control for confounding	
7			
Statistical methods	#12b	Describe any methods used to examine subgroups and interactions	7
Statistical methods	#12c	Explain how missing data were addressed	7
Statistical methods	#12d	If applicable, explain how loss to follow-up was addressed	7
Statistical methods	#12e	Describe any sensitivity analyses	
7			

Results

Participants	#13a	Report numbers of individuals at each stage of study—eg numbers potentially eligible, examined for eligibility, confirmed eligible,	5

For peer review only

		included in the study, completing follow-up, and analysed. Give information separately for for exposed and unexposed groups if applicable.	
Participants	#13b	Give reasons for non-participation at each stage	5
Participants	#13c	Consider use of a flow diagram	
5			
Descriptive data	#14a	Give characteristics of study participants (eg demographic, clinical, social) and information on exposures and potential confounders. Give information separately for exposed and unexposed groups if applicable.	7
Descriptive data	#14b	Indicate number of participants with missing data for each variable of interest	
7			
Descriptive data	#14c	Summarise follow-up time (eg, average and total amount)	
7			
Outcome data	#15	Report numbers of outcome events or summary measures over time. Give information separately for exposed and unexposed groups if applicable.	
7			
Main results	#16a	Give unadjusted estimates and, if applicable, confounder-adjusted estimates and their precision (eg, 95% confidence interval). Make clear which confounders were adjusted for and why they were included	9
Main results	#16b	Report category boundaries when continuous variables were categorized	7
Main results	#16c	If relevant, consider translating estimates of relative risk into absolute risk for a meaningful time period	
8			
Other analyses	#17	Report other analyses done—eg analyses of subgroups and interactions, and sensitivity analyses	9

Discussion

For peer review only

Key results	#18	Summarise key results with reference to study objectives	10
Limitations	#19	Discuss limitations of the study, taking into account sources of potential bias or imprecision. Discuss both direction and magnitude of any potential bias.	11
Interpretation	#20	Give a cautious overall interpretation considering objectives, limitations, multiplicity of analyses, results from similar studies, and other relevant evidence.	12
Generalisability	#21	Discuss the generalisability (external validity) of the study results	12
**Other Information**			
Funding	#22	Give the source of funding and the role of the funders for the present study and, if applicable, for the original study on which the present article is based	13

The STROBE checklist is distributed under the terms of the Creative Commons Attribution License CC-BY. This checklist was completed on 19. February 2023 using https://www.goodreports.org/, a tool made by the EQUATOR Network in collaboration with Penelope.ai

For peer review only

BMJ Open

Concurrent trajectories of part-time work and sickness absence - a longitudinal cohort study over 11 years among shift working hospital employees

Journal:	*BMJ Open*
Manuscript ID	bmjopen-2023-072987.R1
Article Type:	Original research
Date Submitted by the Author:	17-Aug-2023
Complete List of Authors:	Ropponen, Annina; Finnish Institute of Occupational Health; Karolinska Institute, Department of Clinical Neuroscience<br>Ervasti, Jenni; Finnish Institute of Occupational Health, Topeliuksenkatu 41 a A, FI-00250, Helsinki, Finland<br>Harma, Mikko; Finnish Institute of Occupational Health, Work Ability and Work Careers
**Primary Subject Heading**:	Epidemiology
Secondary Subject Heading:	Occupational and environmental medicine
Keywords:	EPIDEMIOLOGY, Human resource management < HEALTH SERVICES ADMINISTRATION & MANAGEMENT, OCCUPATIONAL & INDUSTRIAL MEDICINE

SCHOLARONE™
Manuscripts

I, the Submitting Author has the right to grant and does grant on behalf of all authors of the Work (as defined in the below author licence), an exclusive licence and/or a non-exclusive licence for contributions from authors who are: i) UK Crown employees; ii) where BMJ has agreed a CC-BY licence shall apply, and/or iii) in accordance with the terms applicable for US Federal Government officers or employees acting as part of their official duties; on a worldwide, perpetual, irrevocable, royalty-free basis to BMJ Publishing Group Ltd ("BMJ") its licensees and where the relevant Journal is co-owned by BMJ to the co-owners of the Journal, to publish the Work in this journal and any other BMJ products and to exploit all rights, as set out in our licence.

The Submitting Author accepts and understands that any supply made under these terms is made by BMJ to the Submitting Author unless you are acting as an employee on behalf of your employer or a postgraduate student of an affiliated institution which is paying any applicable article publishing charge ("APC") for Open Access articles. Where the Submitting Author wishes to make the Work available on an Open Access basis (and intends to pay the relevant APC), the terms of reuse of such Open Access shall be governed by a Creative Commons licence – details of these licences and which Creative Commons licence will apply to this Work are set out in our licence referred to above.

Other than as permitted in any relevant BMJ Author's Self Archiving Policies, I confirm this Work has not been accepted for publication elsewhere, is not being considered for publication elsewhere and does not duplicate material already published. I confirm all authors consent to publication of this Work and authorise the granting of this licence.

For peer review only

Concurrent trajectories of part-time work and sickness absence - a longitudinal cohort study over 11 years among shift working hospital employees

Authors: Annina Ropponen[1,2], Jenni Ervasti[1], Mikko Härmä[1]

[1] Finnish Institute of Occupational Health, Helsinki, Finland

[2] Division of Insurance Medicine, Department of Clinical Neuroscience, Karolinska Institute, Stockholm, Sweden

Corresponding author: Annina Ropponen, Finnish Institute of Occupational Health, P.O. Box 18, 00032 Finnish Institute of Occupational Health, Finland, email. annina.ropponen@ttl.fi

Word count: 2627.

For peer review only

Abstract

Objectives: To investigate the concurrent changes in part-time work and sickness absence (SA) in healthcare. Another aim was to investigate the role of age and sex on different concurrent trajectory groups.

Design: Prospective cohort study.

Setting: Public hospital districts (n = 10) and cities (n = 11) in Finland.

Participants: Payroll-based objective working hour data of healthcare sector in Finland for 28969 employees in 2008-2019 were utilized. The final sample included those working shifts with three consecutive years of data and without baseline (≥14 days) SA.

Primary outcomes: Part-time work (yes or no) and months of SA.

Measures: Group-based trajectory modelling to identify concurrent changes in part-time work, and months of SA while controlling time-variant amount of night work and multinomial regression models for relative risk (RR) with 95% confidence intervals (CI) were used.

Results: Four-group trajectory model was the best solution: Group 1 (61.2%) with full-time work and no SA, Group 2 (16.9%) with slowly increasing probability of part-time work and low but mildly increasing SA, Group 3 (17.6%) with increasing part-time work and no SA, and Group 4 (4.3%) with fluctuating, increasing part-time work and highest and increasing levels of SA. Men had a lower (RR 0.49-0.75), and older age groups had a higher likelihood (RRs 1.32-3.79) of belonging to trajectory groups 2-4.

Conclusions Most of the sample were in the trajectory group having full-time work and no SA. Probability of part-time work increased over time being linked with concurrent low increase or no SA. A minor group of employees had both an increased probability of part-time work and SA. Part-time work, but also other solutions might merit attention to promote sustainable working life among healthcare employees.

For peer review only

Article summary

Strengths and limitations of this study

- Payroll-based register data of working hours enabled objective assessment of part-time work and sickness absence.
- Homogenous sample of hospital employees working in specialized healthcare services or at primary healthcare, including e.g., health centers, and local hospitals for elderly and chronic ill people providing 24/7 care, or home care for disabled.
- Group-based trajectory model (GBTM) to identify concurrent trajectories of part-time work and mean number of sickness absence months/years for the follow-up from 2009 to 2019 to estimate a distinct pattern over time.
- Lack of information on the reasons for part-time work and no information of diagnosis or part-time sickness absence.

Keywords: shift work, part-time work, hospital, sick leave, longitudinal study

For peer review only

Background

Healthcare sector is facing major challenges due to population ageing, increased need for care, and economic challenges combined with staff shortage. The ageing population affects both ensuring sufficient levels of nursing staff, but also the growing demands for care of the ageing population [1, 2]. The existing need for longer work careers [3] combined with increasing turnover rates emphasizes the urgent need to understand working hours in association with work capacity and sustainable working life. This calls for understanding for associations between working hours and work capacity and builds on the sustainable work which is “working and living conditions that are such that they support people in engaging and remaining in work throughout an extended working life” [4]. For example, in recent years sickness absences (around 10% of all employed) have been in increase in Finland[5] and especially at healthcare [6]. In shift work, two working hour solutions may provide a possibility to decrease workload due to demanding working conditions, or to arrange more time for care for small children or other dependents: an employee may work part-time, or to change from night work to day-time work [7, 8]. However, part-time work may also be health-related, i.e., being based on part-time sickness absence or disability pension [9, 10]. Until now, studies on the role of part-time work in relation future long-time trajectories of sickness absence are lacking. As sickness absence increases costs both to the employers and the society, further understanding of the linkage with part-time work is needed.

Working part-time may, besides influencing the income, have long-term consequences on career and pension [11]. In Finland, every fifth woman (13% men) work part-time [12]. On the other hand, shorter working hours and increased time for recovery could lengthen the work careers [13]. Due to an emergent need to increase employment rates and promote sustainable working life among the working population [14, 15], longitudinal studies investigating the role of part-time work are important. Many studies have reported associations between individual sociodemographic, socioeconomic, health- and work-related factors including part-time work for labor market exit due

For peer review only

to sickness absence or disability pension [16, 17]. However, until now, studies with longitudinal designs to investigate associations between part-time work and sustainable working life have mainly been based on survey data [15, 18-23].

A hypothesis of this study was that due to ageing, older employees may choose part-time work due to decreased health (reflected in higher sickness absence) more often than younger employees. We also hypothesize that part-time work without increased sickness absence might be more frequent among younger employees than older ones e.g., due to need to combine work and life while having small children or with studies. The possibility to combine part-time work with part-time sickness absence, that is, to shorten working hours when having health problems and while compensated by social benefits (i.e., sickness absence benefits), may extend and/or maintain the working lives of the older employees [24]. However, part-time work and sickness absence may also both increase along age. Another aspect related to age is night work, i.e., older employees may wish to work more during the days than nights [25]. Also age-related trends in the associations of night work and health have been identified [26, 27]. Therefore, based on the assumption that younger and older employees might react differently to night work [28, 29], one should control night work when investigating age-related differences in part-time work and sickness absence. This is relevant, especially in longitudinal settings in which working during the nights may decrease due to possibilities to select more favourable working hours [30, 31].

This study aimed to investigate the concurrent changes in part-time work and sickness absence among employees in healthcare. Another aim was to investigate the role of age and sex on different concurrent trajectory groups.

Methods

This study was based on the ongoing Working Hours in the Finnish Public Sector study (WHFPS) [32, 33] with payroll-based register data of working hours for 2008-2019. To ensure follow-up across years and to exclude those with exceptionally long absences (due to sickness, parental leave, or

For peer review only

other reasons) and very short working periods, we restricted the sample to those with ≥31 work shifts/year in three consecutive years during 2008-2019. Then the sample was restricted to those without sickness absence (≥14 consequent days) at baseline in 2008. Last, only those with shift work contract were included (Figure 1). The final sample was 28969 shift working employees in 10 hospital districts and 11 cities using Titania® shift scheduling software. In Finland, the hospital districts (45% of the final sample) are responsible for specialized healthcare services providing both specialized outpatient- and inpatient care. The healthcare employees in the cities (55% of the final sample) are those working at primary healthcare, including e.g., health centers, and local hospitals for elderly and chronic ill people providing 24/7 care, or home care for disabled [33].

In this study, we utilized annual working hour and sickness absence data from 2008-2019 as has been described in detail before [32, 33]. Part-time work was based on work contract information and coded yes or no for each year based on annual median. Then annual sickness absence of any length was measured in months/year for follow-up from 2009 to 2019. For the baseline 2008, the short sickness absences (≤3 days) were estimated. We also utilized the proportion of night work of all work shifts/year to estimate the time-varying effect of night work as a proxy of shift work in this study. Since aging employees may change from night work to day work [25, 34, 35], the proportion of night work was assumed to reflect this change, which in turn might affect the rate of part-time work. Baseline age (in 2008 and years categorized into < 25 years of age, ≥25 and <40 years, ≥40 and <55 years, and >55 years based on distribution), and sex were used in the analyses.

Patient ja public involvement

Patients or the public were not involved in the design, or conduct, or reporting, or dissemination plans of our research.

Statistical analyses

First, we calculated the descriptive characteristics for the study sample. Then using a group-based multi-trajectory model, we identified concurrent trajectories of part-time work and mean number of

For peer review only

sickness absence months/years for the follow-up from 2009 to 2019 while accounting night work as time varying covariate [36]. The group-based multi-trajectory method enables to identify groups of individuals (trajectory groups) which follow a distinct pattern over time. The linear polynomial model was applied. Bayesian information criterion (BIC), Akaike Information Criteria (AIC) and average posterior probability (APP) were used to determine the best-fitting model. Third, we ran multinomial regression models for relative risk (RR) and 95% Confidence Intervals (CI) using sex and age groups for predicting the group membership for each trajectory group. All statistical analyses were conducted with Stata 17.1 MP.

Results

At baseline in 2008, 7% of the final sample of 28969 shift working employees worked part-time, mean age was 39.8 years (SD 10.7), 92% were women, and 34% had had at least one short sickness absence (≤3 days). The amount of night shifts was 8% of all shifts. During the follow-up (from 2009 to 2019), the mean number of sickness absence months varied from 0.3 months in 2009 to 0.6 months in 2018 (range 0-12 months).

A four-cluster model was chosen based on BIC and AIC values and parsimony for concurrent changes in part-time work and months of sickness absence (Table 1).

Table 1 Goodness of fit statistics of group-based trajectory analysis models

	Smallest group				
	N	%	BIC	AIC	APP
2-cluster model	4204	16.5	-190414.9	-190352.8	0.90
3-cluster model	3795	14.4	-181308.3	-181213.1	0.89
**4-cluster model***	**1099**	**4.3**	**-179517.1**	**-179388.9**	**0.87**
5-cluster model	1156	4.4	-175817.0	-175655.6	0.86
6-cluster model	1134	4.4	-175451.2	-175256.7	0.86
7-cluster model	688	3.7	-17117.5	-173890.0	0.79

* The models presented are shown in bold. BIC = Bayesian Information Criterion, AIC = Akaike Information Criterion, and APP = average posterior probability.

The concurrent trajectories were (Figure 2):

1. Group 1 (61.2% of the final sample) with full-time work and no sickness absence.

For peer review only

2. Group 2 (16.9) with slowly increasing probability of part-time work and low but mildly increasing sickness absence.
3. Group 3 (17.6%) with increasing part-time work and no sickness absence.
4. Group 4 (4.3%) with fluctuating but increasing part-time work and highest and increasing levels of sickness absence.

The regression models (Table 2) showed that compared to the youngest age group (<25 years of age at the baseline), the older age groups had increased likelihood of belonging to the trajectory groups 2-4 (in comparison to trajectory group 1). Men had lower likelihood of belonging to trajectory groups 2-4 than women (Table 2).

Table 2 Sex and age group associated with cluster membership (part-time work and months of sickness absence). Multinomial regression (ref=Cluster 1 'Full-time work and no sickness absence', n=2124). Relative risk ratio (RR) with 95% confidence intervals (CI)

	**Group 2** Slowly increasing probability of part-time work and low but mildly increasing sickness absence		**Group 3** Increasing part-time work and no sickness absence		**Group 4** Fluctuating but increasing part-time work and highest and increasing levels of sickness absence	
	**RR***	**95%CI**	**RR**	**95%CI**	**RR**	**95%CI**
Women	1		1		1	
Men	**0.74**	**0.63, 0.86**	**0.75**	**0.65, 0.87**	**0.49**	**0.34, 0.71**
Age <25 years	1		1		1	
25-39 years	**1.32**	**1.13, 1.54**	**1.56**	**1.36, 1.80**	**2.36**	**1.56, 3.56**
40-54 years	**1.83**	**1.58, 2.12**	1.10	0.95, 1.26	**3.79**	**2.54, 5.66**
≥55 years	**1.69**	**1.39, 2.07**	**1.53**	**1.27, 1.85**	**2.99**	**1.83, 4.88**

* Statistically significant RR with 95%CI in boldface

In the supplemental material, we present the concurrent trajectories without adjusting for time-variant night work (Supplemental Figure 1), and for each age group separately (Supplemental Figures S2-5) and their fit indexes in the supplemental tables S1-2. Although trajectory groups 1-3 are similar than adjusting for time-variant night work, the group 4 indicates some changes in the probability of part-time work. Except for those aged 25-39 years with four trajectory groups, all other age groups

For peer review only

were best fitted to a five-group trajectory solution. The concurrent changes of part-time work and sickness absence varied between age groups: among the oldest age group, ≥55 years, three out of four trajectory groups had increase in part-time work associated with low or no sickness absence. The concurrent trajectories with decrease in part-time work (transition to full-time) with no sickness absence were identified among middle age groups (25-54 years).

Discussion

This longitudinal study of nearly 29 000 Finnish employees in healthcare sector investigated the concurrent changes in part-time work and sickness absence across 11 years, and the role of age and sex on the identified concurrent trajectories. Four trajectory groups were detected: Group 1 (61%) with full-time work and no sickness absence, Group 2 (17%) with slowly increasing probability of part-time work and low but mildly increasing sickness absence, Group 3 (18%) with increasing part-time work and no sickness absence, and Group 4 (4%) with fluctuating but increasing part-time work and highest and increasing levels of sickness absence. Women and older employees were more likely to belong to groups 2-4 characterized by increasing part-time work. This is among the first studies to investigate the concurrent changes of part-time work and sickness absence from 2009 to 2019 while controlling for night work among healthcare employees. Hence, this study adds to the earlier knowledge based on survey data [15, 18-23].

The largest trajectory group (61%) worked full-time and had no sickness absence reflecting the well-known trend of women working mostly full-time in the Nordic countries [11]. However, we also detected two moderate size groups (trajectory groups 2 and 3) with increasing probability of part-time work and low but mildly increasing, or no sickness absence. These might be indicative for our hypotheses that a) along ageing, older employees may choose part-time work due to decreasing health (with higher sickness absence), or b) part-time work with low or no sickness absence might appear among younger employees due to needs to balance work and life. Since the fourth trajectory group, although small (4%), indicated fluctuating, but increasing part-time work and highest and

For peer review only - http://bmjopen.bmj.com/site/about/guidelines.xhtml

increasing sickness absence, it seems to support the above-mentioned hypothesis, and the assumption that both part-time work and sickness absence increase along age. Our Supplemental figure S1 confirmed that overall, four group trajectory solution was the best even without time variant night work. However, when night work was not accounted for (Supplemental figure S1), the probability of part-time work was less likely indicating that transition to day work may be another strategy to affect the working hours along age [25]. This might be an important notion for workplaces and occupational healthcare since age-related trends in the associations of night work and health exist [26, 27] and perhaps modifications to working hours could promote sustainable working life [14, 15] if part-time work would be compensated with social benefits.

We were specifically interested on the role of sex and age on these concurrent changes of part-time work and sickness absence. Women had a higher likelihood of belonging to trajectory groups 2-4 characterized by increases in part-time work and with or without sickness absence. This is in line with earlier findings indicating that e.g., a typical employee having long-term sickness absence is a woman at her 50's and working in the healthcare sector [37, 38]. This might be because women have higher sickness absence rates than men [39-41], but also because women outnumber men in the healthcare sector (in our study, only 8% were men). Another influential, well-known fact is that men and women work part-time due to different reasons such as child care or health [11] and even societal factors such as availability of daycare for children or salary levels can play a role [42-44]. Older age groups had a higher likelihood of belonging to trajectory groups 2-4 compared to group 1. There are some possible explanations. First, the concurrent increase of part-time work and full-time sickness absence along age. Alternatively age-related trends of reacting to night work [28, 29], i.e., across age groups the concurrent changes of part-time work and sickness absence were different as shown in our supplement figures. Thus, further studies would be needed to address the various reasons of part-time work (e.g., health, availability of work), health issues (diagnoses for sickness absence), but also more detailed time periods (e.g., monthly, or weekly) and working hour-based measures of part-time work. In addition, non-observational research designs are also needed.

For peer review only

We had access to objective working hour data including sickness absence from 2008 to 2019 almost in total 240000 Finnish employees in healthcare sector. This unique dataset enabled us to restrict the sample to those working shift work, having complete data for ≤3 consecutive years during 2009-2019, and without baseline ≥14 days sickness absence. These are evident strengths although the final sample size was around 29000 employees. Such data is free from reporting or memory bias adding to earlier knowledge based on mainly survey data [15, 18-23]. However, a limitation is that we had no information on the reasons for part-time work which may be due to e.g., health, personal matters, or unavailability of full-time work [45, 46]. We also cannot rule out the healthy worker bias but assume it might not to play a major role. Furthermore, we only had information of sickness absence without diagnosis and no information on part-time sickness absence. Since part-time work may relate to health status, i.e., being based on part-time sickness absence or disability pension [9, 10], detailed health information should be accounted in further studies. We also lacked information on many sociodemographic factors (i.e., marital status, number of children, level of income) which is a limitation. Further studies should be designed to address these factors. As in any observational study, we were unable to demonstrate the causality between part-time work and sickness absence. It may be that transfer into part-time work indicates health problems and thus is a marker or a risk factor for subsequent sickness absence. However, part-time work may also be a steppingstone when returning to work after sickness absence. Future research on the interrelations between these two phenomena is needed. Yet another limitation might be generalizability of the findings. In Finland, and in Nordic countries, work at healthcare is organized mostly via irregular working hours, i.e., shift work with a non-standard working hour schedule with variation in start and finish times, lengths, and rest periods between work shifts [47]. On the other hand, sickness absence is subsidized via welfare society model typical to Nordic countries. Hence our findings might be more generalizable to Nordic than other countries.

Conclusions

For peer review only

The majority (61%) of employees worked full-time and without sickness absence throughout the 11-year follow-up. However, for the rest of the employees, the probability of part-time work increased over time with varying level of simultaneous changes in sickness absence. Increase in part-time work was more probable for women and for older employees. For a minority of the employees (4%), the increase in part-time work was strongly linked with concurrent increase in sickness absence. The relationship between part-time work and sickness absence is likely bidirectional. Transfer into part-time work may indicate health problems and subsequent sickness absence, but part-time work may also be used when gradually returning to work after sickness absence and thus it can promote sustainable working life in healthcare employees.

For peer review only

Statements and declarations

Acknowledgements

This work was supported by the Academy of Finland, DIGIHUM-programme grant number 329200.

Conflict of interest

The authors declare that they have no conflict of interest.

Author contributions

AR, MH, and JE contributed to the study's conception and design. Material preparation, data collection and analysis were performed by AR. The first draft of the manuscript was written by AR, and all the authors commented on previous versions of the manuscript. All the authors read and approved the final manuscript.

Availability of data and materials

The data that support the findings of this study are available from the Hospital districts and Cities but restrictions apply to the availability of these data, which were used under license for the current study, and so are not publicly available. Data are however available from the last author upon reasonable request and with permission of the Hospital districts and Cities.

Ethics approval

This study was fully based on employer-owned administrative employment register data that the Hospital Districts and Cities had permitted the access and applied pseudonymized identification numbers for research purposes. Using such data does not include any experimental protocols requiring approval. In Finland, research using such data does not need to undergo review by an ethics committee according to national legislation (Medical Research Act).

Consent to participate

For peer review only

The consent to participate was not applicable since the data comprised employer-owned employment information and was analyzed as administrative register data pseudonymized for research purposes.

Consent for publication

Not applicable.

For peer review only

Figure legends

Figure 1 Sample selection

Figure 2 Four clusters of trajectories of part-time work and months of sickness absence adjusted for time-variant night work (95% CI are shown as dotted lines, but poorly visible due to being very narrow)

For peer review only

References

1. Tursunbayeva A. Human resource technology disruptions and their implications for human resources management in healthcare organizations. *BMC Health Serv Res* 2019;19(1):268. doi: 10.1186/s12913-019-4068-3 [published Online First: 2019/05/01]
2. Smiley RA, Lauer P, Bienemy C, et al. The 2017 National Nursing Workforce Survey. *Journal of Nursing Regulation* 2018;9(3):S4-S88. doi: Doi 10.1016/S2155-8256(18)30131-5
3. Vanroelen C. Longer working careers and sustainable work: the issue of social inequality. *Society Health & Vulnerability* 2017;8(sup1):1332854. doi: Unsp 1332854

10.1080/20021518.2017.1332854
4. Eurofound. Sustainable working over the life course: concept paper. Publications Office of the European Union, Luxembourg, 2015.
5. Kela. Information package: Sickness absence 2023 [Available from: https://tietotarjotin.fi/en/information-package/2699253/information-package-sickness-absence accessed 1st of August 2023.
6. Health FIoO. Sickness absence in Finnish municipalities 2019 [Available from: https://www.tyoelamatieto.fi/en/dashboards/kunta10-sick-leave accessed 1st of August 2023.
7. Steinmetz S, de Vries DH, Tijdens KG. Should I stay or should I go? The impact of working time and wages on retention in the health workforce. *Hum Resour Health* 2014;12:23. doi: 10.1186/1478-4491-12-23 [published Online First: 2014/04/25]
8. Flodgren GM, Bidonde J. Effects and experiences of part-time work in the health- and community-care services: a scoping review. (Effekter og erfaringer av deltidsarbeid i helse- og omsorgstjenesten: en kartleggingsoversikt). Oslo: National Institute of Public Health, 2019.
9. Schram JL, Solovieva S, Leinonen T, et al. The influence of occupational class and physical workload on working life expectancy among older employees. *Scandinavian journal of work, environment & health* 2021;47(1):5-14. doi: 10.5271/sjweh.3919 [published Online First: 2020/09/01]
10. Ervasti J, Kausto J, Koskinen A, et al. Labor Market Participation Before and After Long-Term Part-Time Sickness Absence in Finland: A Population-Based Cohort Study. *Journal of Occupational and Environmental Medicine* 2020;62(4)
11. NIKK. Faktablad: Vem jobbar och när? Norway: Nordiska ministerrådet, 2018.
12. OECD. Part-time employment rate (indicator) 2023 [accessed 1st of August 2023.
13. Härmä MI, Ilmarinen JE. Towards the 24-hour society--new approaches for aging shift workers? *Scand J Work Environ Health* 1999;25(6):610-5. doi: 10.5271/sjweh.488 [published Online First: 2000/07/07]
14. Health MoSAa. Eheä yhteiskunta ja kestävä hyvinvointi Sosiaali- ja terveysministeriön tulevaisuuskatsaus. Valtioneuvoston julkaisusarja 22/2018. Helsinki: Sosiaali- ja terveysministeriö, 2018.
15. de Wind A, van der Pas S, Blatter BM, et al. A life course perspective on working beyond retirement-results from a longitudinal study in the Netherlands. *BMC Public Health* 2016;16:499. doi: 10.1186/s12889-016-3174-y [published Online First: 2016/06/12]
16. Reinholdt S, Upmark M, Alexanderson K. Health-selection mechanisms in the pathway towards a disability pension. *Work* 2010;37(1):41-51. doi: 10.3233/WOR-2010-1055 [published Online First: 2010/09/23]
17. Ropponen A, Alexanderson K, Svedberg P. Part-time work or social benefits as predictors for disability pension: a prospective study of Swedish twins. *Int J Behav Med* 2014;21(2):329-36. doi: 10.1007/s12529-013-9303-4 [published Online First: 2013/03/13]
18. Lu W, Benson R, Glaser K, et al. Relationship between employment histories and frailty trajectories in later life: evidence from the English Longitudinal Study of Ageing. *J Epidemiol*

Community Health 2017;71(5):439-45. doi: 10.1136/jech-2016-207887 [published Online First: 2016/12/04]

19. Sohier L, Van Ootegem L, Verhofstadt E. Well-Being During the Transition from Work to Retirement. *Journal of Happiness Studies* 2021;22(1):263-86. doi: 10.1007/s10902-020-00228-6
20. Komp-Leukkunen K. Life-histories in Finland: First results from the Survey of Health, Ageing and Retirement in Europe Working papers. Helsinki: Population Research Institute, 2021.
21. Ek E, Ala-Mursula L, Velázquez RG, et al. Employment trajectories until midlife associate with early social role investments and current work-related well-being. *Advances in Life Course Research* 2021;47:100391. doi: https://doi.org/10.1016/j.alcr.2020.100391
22. McDonough P, Worts D, Corna LM, et al. Later-life employment trajectories and health. *Advances in Life Course Research* 2017;34:22-33. doi: https://doi.org/10.1016/j.alcr.2017.09.002
23. de Wind A, Scharn M, Geuskens GA, et al. Predictors of working beyond retirement in older workers with and without a chronic disease - results from data linkage of Dutch questionnaire and registry data. *BMC Public Health* 2018;18(1):265. doi: 10.1186/s12889-018-5151-0 [published Online First: 2018/02/20]
24. Kausto J, Viikari-Juntura E, Virta LJ, et al. Effectiveness of new legislation on partial sickness benefit on work participation: a quasi-experiment in Finland. *BMJ Open* 2014;4(12):e006685. doi: 10.1136/bmjopen-2014-006685
25. Härmä M. Workhours in relation to work stress, recovery and health. *Scand J Work Environ Health* 2006;32(6):502-14.
26. Booker LA, Magee M, Rajaratnam SMW, et al. Individual vulnerability to insomnia, excessive sleepiness and shift work disorder amongst healthcare shift workers. A systematic review. *Sleep Med Rev* 2018;41:220-33. doi: 10.1016/j.smrv.2018.03.005 [published Online First: 2018/04/24]
27. Ritonja J, Aronson KJ, Matthews RW, et al. Working Time Society consensus statements: Individual differences in shift work tolerance and recommendations for research and practice. *Ind Health* 2019;57(2):201-12. doi: 10.2486/indhealth.SW-5 [published Online First: 2019/02/01]
28. Cai C, Vandermeer B, Khurana R, et al. The impact of occupational shift work and working hours during pregnancy on health outcomes: a systematic review and meta-analysis. *Am J Obstet Gynecol* 2019;221(6):563-76. doi: 10.1016/j.ajog.2019.06.051 [published Online First: 2019/07/06]
29. Stock D, Knight JA, Raboud J, et al. Rotating night shift work and menopausal age. *Hum Reprod* 2019;34(3):539-48. doi: 10.1093/humrep/dey390 [published Online First: 2019/02/13]
30. Hess M, Bauknecht J, Pink S. Working Hours Flexibility and Timing of Retirement: Findings from Europe. *J Aging Soc Policy* 2018;30(5):478-94. doi: 10.1080/08959420.2018.1500857 [published Online First: 2018/08/02]
31. Vanajan A, Bultmann U, Henkens K. Health-related Work Limitations Among Older Workers-the Role of Flexible Work Arrangements and Organizational Climate. *The Gerontologist* 2019 doi: 10.1093/geront/gnz073 [published Online First: 2019/06/01]
32. Harma M, Ropponen A, Hakola T, et al. Developing register-based measures for assessment of working time patterns for epidemiologic studies. *Scand J Work Environ Health* 2015;41(3):268-79. doi: 10.5271/sjweh.3492 [published Online First: 2015/03/20]
33. Härmä MI, Shiri R, Ervasti J, et al. National Recommendations for Shift Scheduling in Healthcare: A 5-Year Prospective Cohort Study on Working Hour Characteristics. 2022 doi: http://dx.doi.org/10.2139/ssrn.4084020
34. Blok MM, de Looze MP. What is the evidence for less shift work tolerance in older workers? *Ergonomics* 2011;54(3):221-32. doi: 10.1080/00140139.2010.548876

For peer review only

35. Härmä M, Karhula K, editors. *Working hours, health, well-being and participation in working life : Current knowledge and recommendations for health and safety*. Helsinki, Finland: Työterveyslaitos, 2020.
36. Nagin DS, Jones BL, Passos VL, et al. Group-based multi-trajectory modeling. *Stat Methods Med Res* 2018;27(7):2015-23. doi: 10.1177/0962280216673085 [published Online First: 2018/05/31]
37. Försäkrings A. Psykisk ohälsa i olika län, 2019.
38. samhällsmedicin Cfeo. Coronapandemin och psykisk hälsa. En prognos om kort- och långsiktiga effekter. Stockholm: Region Stockholm, 2020.
39. Borg K, Hensing G, Alexanderson K. Risk factors for disability pension over 11 years in a cohort of young persons initially sick-listed with low back, neck, or shoulder diagnoses. *Scand J Public Health* 2004;32(4):272-8. doi: 10.1080/14034940310019524
40. Haukenes I, Gjesdal S, Rortveit G, et al. Women's higher likelihood of disability pension: the role of health, family and work. A 5-7 years follow-up of the Hordaland Health Study. *BMC public health* 2012;12:720. doi: 10.1186/1471-2458-12-720
41. Mykletun A, Overland S, Dahl AA, et al. A population-based cohort study of the effect of common mental disorders on disability pension awards. *Am J Psychiatry* 2006;163(8):1412-8. doi: 10.1176/ajp.2006.163.8.1412 [published Online First: 2006/08/01]
42. Barbieri P, Cutuli G, Guetto R, et al. Part-time employment as a way to increase women's employment: (Where) does it work? *International Journal of Comparative Sociology* 2019;60(4):249-68. doi: 10.1177/0020715219849463
43. EuroStat. Being young in Europe today - labour market - access and participation 2020 [Available from: https://ec.europa.eu/eurostat/statistics-explained/index.php?title=Being_young_in_Europe_today_-_labour_market_-_access_and_participation.
44. Viikari-Juntura E, Leinonen T, Virta LJ, et al. Early part-time sick leave results in considerable savings in social security costs at national level: an analysis based on a quasi-experiment in Finland. *Scand J Work Environ Health* 2019;45(2):203-08. doi: 10.5271/sjweh.3780 [published Online First: 2018/10/20]
45. Pech C, Klainot-Hess E, Norris D. Part-time by Gender, Not Choice: The Gender Gap in Involuntary Part-time Work. *Sociological Perspectives* 2021;64(2):280-300. doi: 10.1177/0731121420937746
46. Rui F, Jennifer G, Peter CC. *Research Square* 2021 doi: 10.21203/rs.3.rs-390046/v1
47. Sallinen M, Kecklund G. Shift work, sleep, and sleepiness - differences between shift schedules and systems. *Scand J Work Environ Health* 2010;36(2):121-33. doi: 10.5271/sjweh.2900 [published Online First: 2010/02/02]

For peer review only

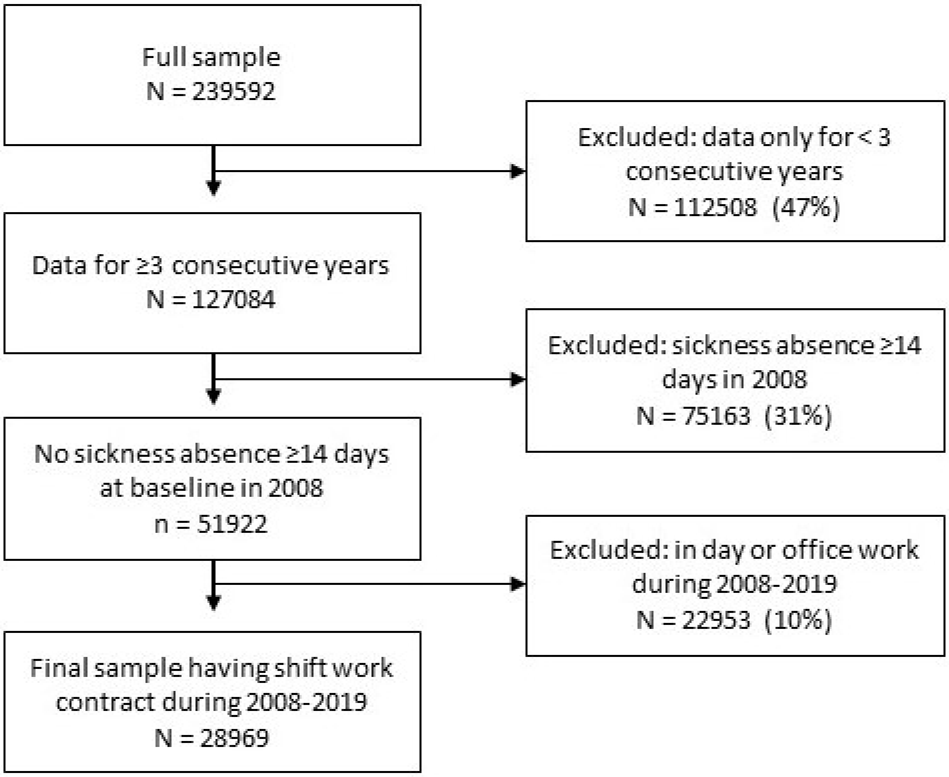

127x104mm (96 x 96 DPI)

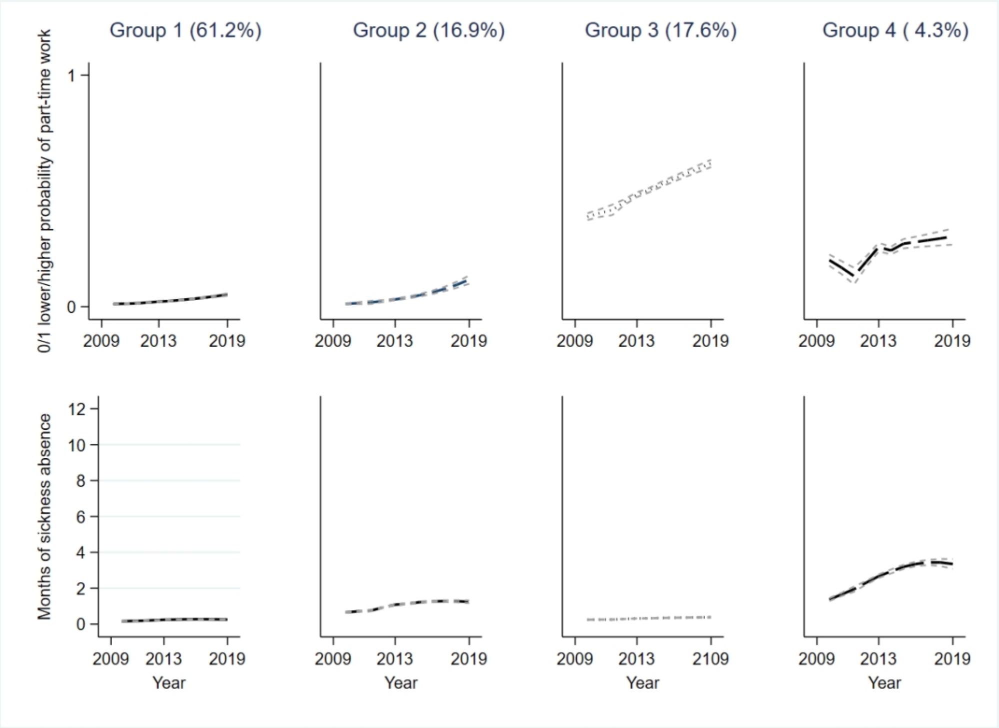

428x312mm (72 x 72 DPI)

Supplemental material

Concurrent trajectories of part-time work and sickness absence - a longitudinal cohort study over 11 years among shift working hospital employees

For peer review only

Supplemental material

Supplemental Table S1 Goodness of fit statistics of group-based trajectory analysis models

	Smallest group				
	**N**	**%**	**BIC**	**AIC**	**APP**
2-cluster model	5461	17	-263123.4	-263077.8	0.92
3-cluster model	5184	16	-254453.0	-254382.6	0.91
**4-cluster model***	**1671**	**5**	**-250141.8**	**-250046.5**	**0.91**
5-cluster model	919	4	-248427.7	-248307.6	0.84
6-cluster model	920	4	-244756.5	-244611.4	0.83

* The models presented are shown in bold. BIC = Bayesian Information Criterion, AIC = Akaike Information Criterion, and APP = average posterior probability.

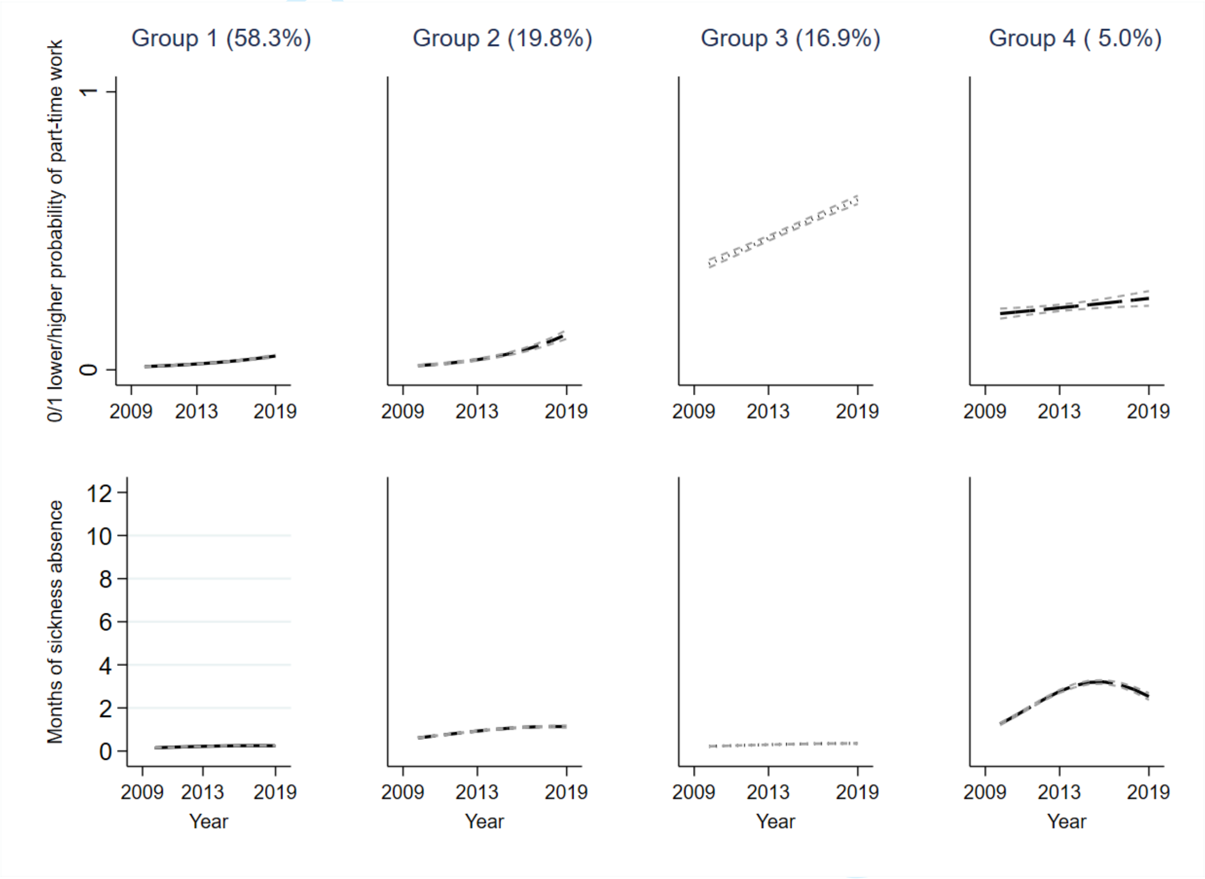

Supplemental Figure S1 Four clusters of trajectories of part-time work and months of sickness absence (95% CI are shown as dotted lines, but poorly visible due to being very narrow)

Supplemental material

Supplemental Table S2 Goodness of fit statistics of group-based trajectory analysis models

	**Baseline age < 25 years (n = 1950)**					**Baseline age ≥25 and <40 years (n = 7437)**				
	**Smallest group**					**Smallest group**				
	**N**	**%**	**BIC**	**AIC**	**APP**	**N**	**%**	**BIC**	**AIC**	**APP**
2-cluster model	453	25	-13732.04	-13690.23	0.90	1790	25	-63056.56	-63004.71	0.93
3-cluster model	235	14	-13234.66	-13170.54	0.88	928	13	-59538.30	-59458.79	0.90
**4-cluster model***	95	6	-13196.39	-13109.97	0.78	**649**	**10**	**-58972.56**	**-58865.39**	**0.86**
**5-cluster model***	**80**	**5**	**-13183.34**	**-13074.62**	**0.83**	274	4	-58496.44	-58361.61	0.88
6-cluster model	70	4	-13142.51	-13011.84	0.81					
7-cluster model	17	1	-13118.99	-12965.66	0.93					
	**Baseline age ≥40 and <55 years (n = 10143)**					**Baseline age ≥55 years (n = 1430)**				
2-cluster model	2626	26	-94282.84	-94228.65	0.94	322	23	-12128.61	-12089.12	0.97
3-cluster model	1120	11	-89502.96	-89419.88	0.94	259	19	-11463.96	-11403.41	0.91
4-cluster model	597	6	-87992.44	-87880.46	0.91	154	10	-11295.51	-11213.90	0.91
**5-cluster model***	**535**	**5**	**-87053.32**	**-86912.44**	**0.91**	**139**	**10**	**-11202.11**	**-11099.44**	**0.84**
6-cluster model	277	3	-86516.63	-86346.85	0.87	54	4	-11094.27	-10970.53	0.89

* The models presented are shown in bold. BIC = Bayesian Information Criterion, AIC = Akaike Information Criterion, and APP = average posterior probability.

For peer review only

Supplemental material

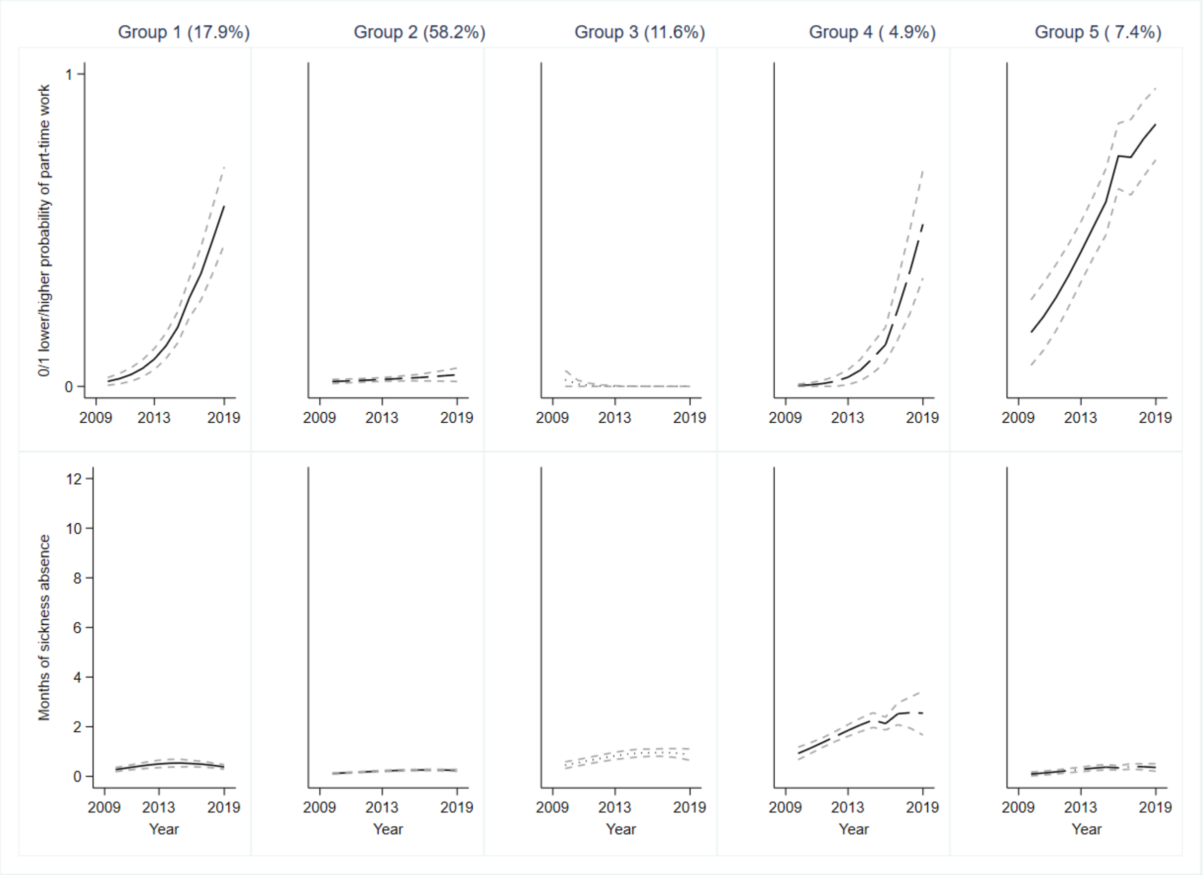

Supplemental Figure S2 Five clusters of trajectories of part-time work and months of sickness absence **among those with baseline age <25 years** (n = 1950) adjusted for time-variant night work (95% CI are shown as dotted lines, but poorly visible due to being very narrow)

Supplemental material

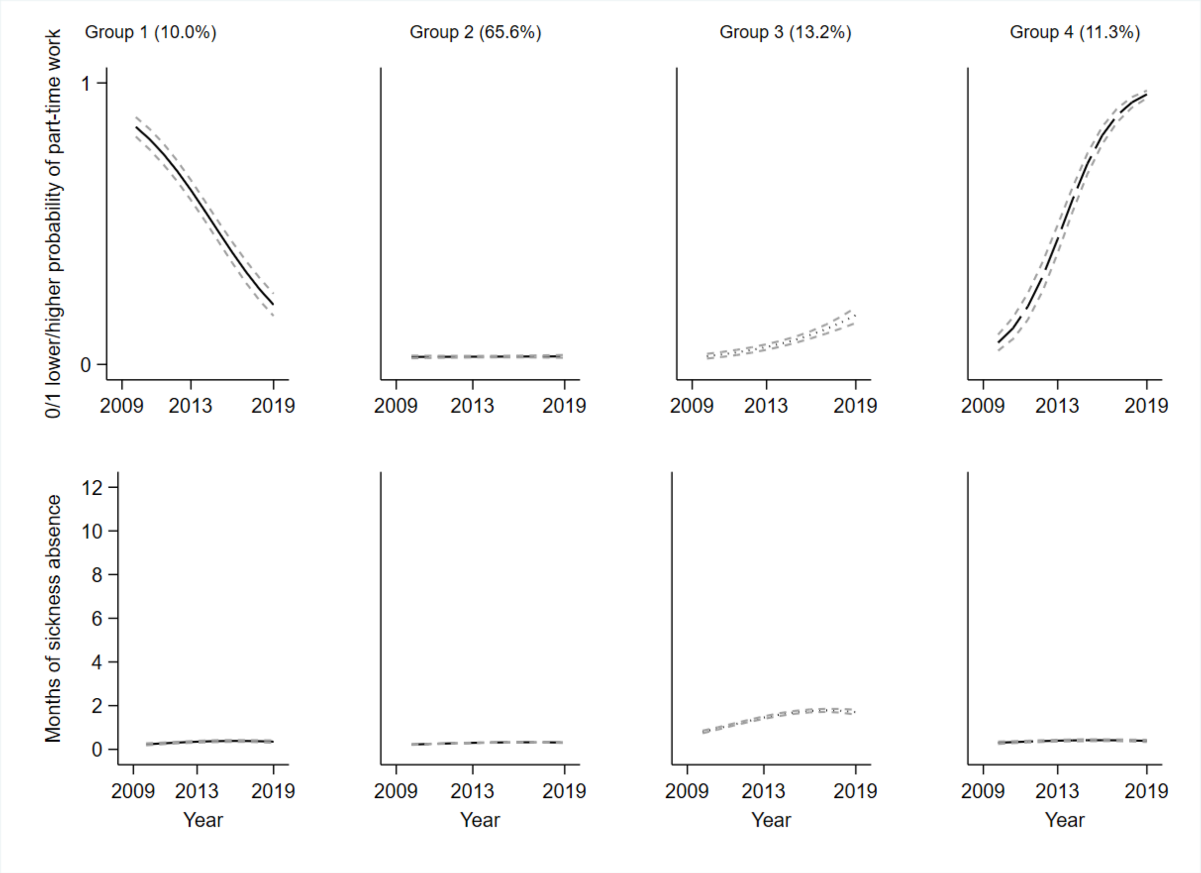

Supplemental Figure S3 Four clusters of trajectories of part-time work and months of sickness absence **among those with baseline age ≥25 and <40 years** (n = 7437) adjusted for time-variant night work (95% CI are shown as dotted lines, but poorly visible due to being very narrow)

For peer review only

Supplemental material

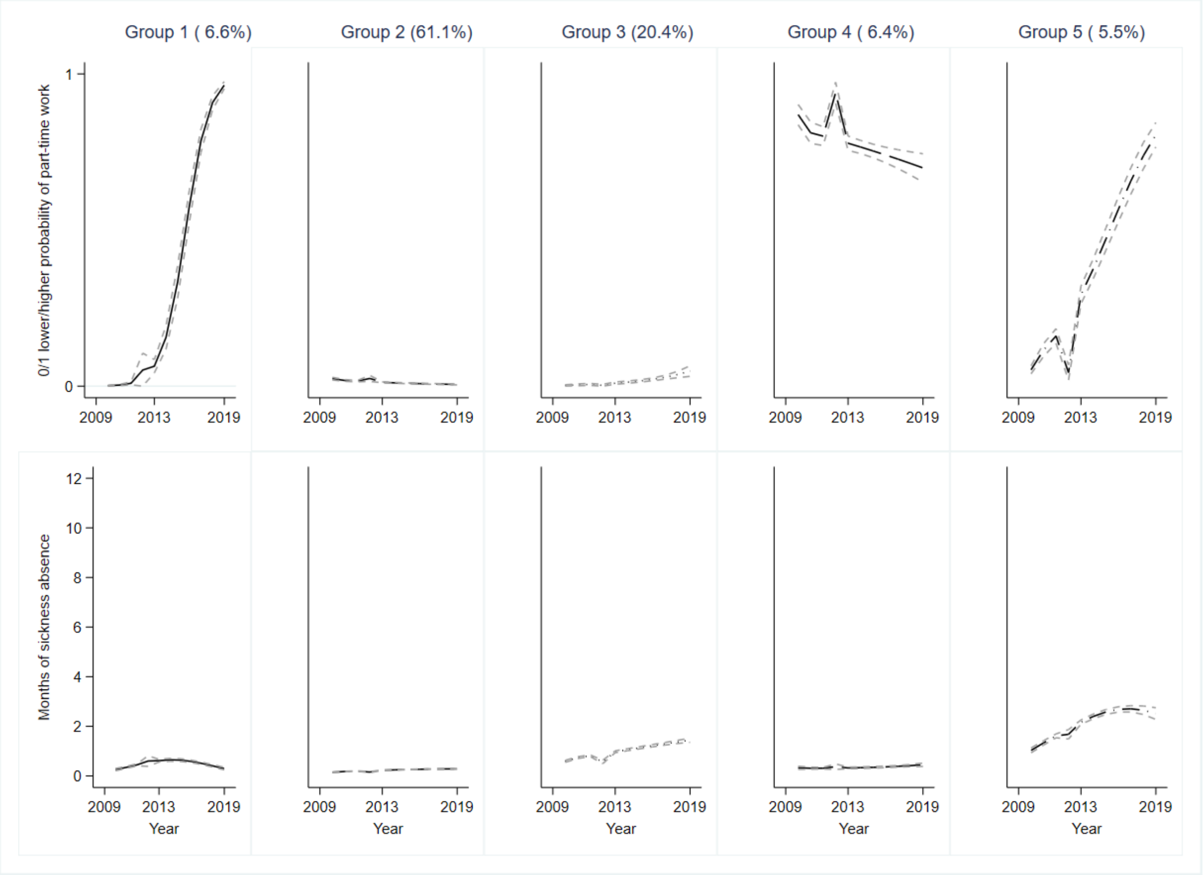

Supplemental Figure S4 Five clusters of trajectories of part-time work and months of sickness absence **among those with baseline age ≥40 and <55 years** (n = 10143) adjusted for time-variant night work (95% CI are shown as dotted lines, but poorly visible due to being very narrow)

Supplemental material

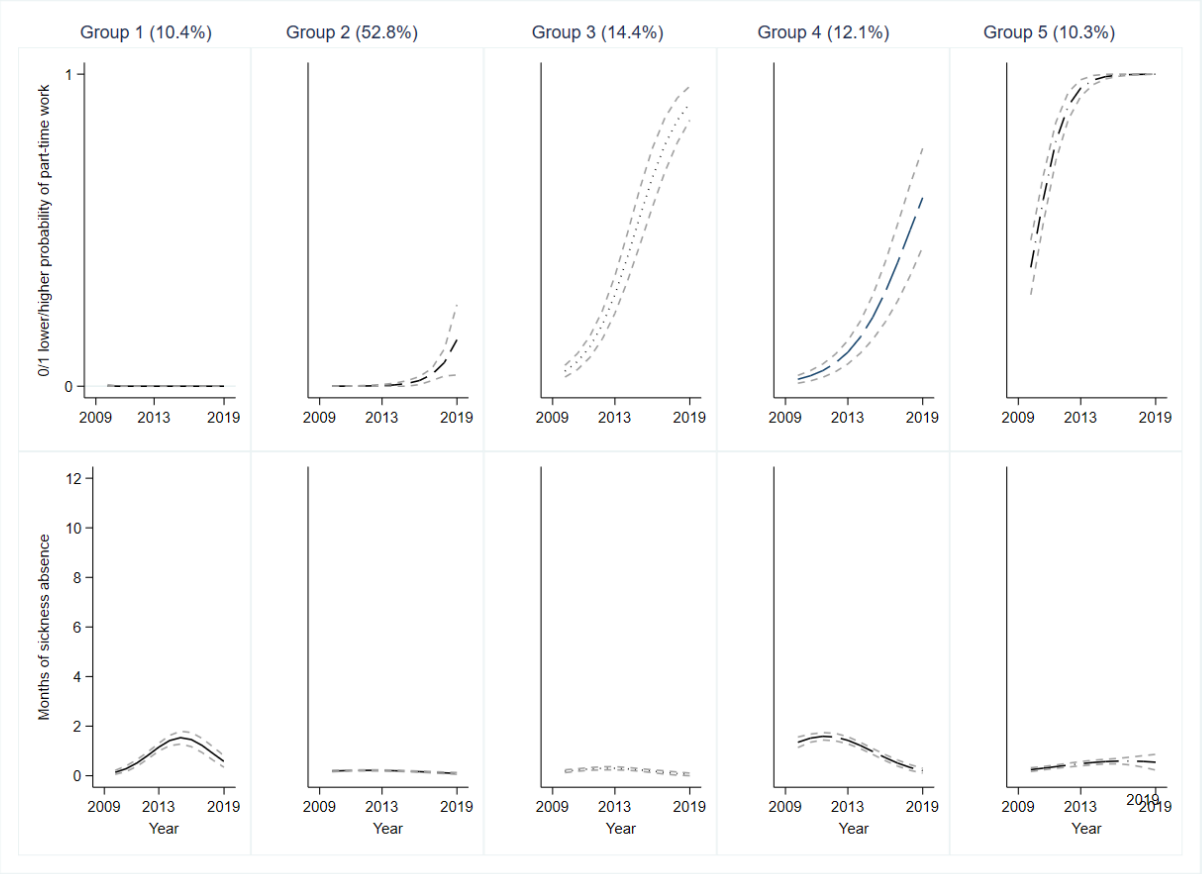

Supplemental Figure S5 Five clusters of trajectories of part-time work and months of sickness absence **among those with baseline age ≥55 years** (n = 1430) adjusted for time-variant night work (95% CI are shown as dotted lines, but poorly visible due to being very narrow)

For peer review only

Reporting checklist for cohort study.

Based on the STROBE cohort guidelines.

Instructions to authors

Complete this checklist by entering the page numbers from your manuscript where readers will find each of the items listed below.

Your article may not currently address all the items on the checklist. Please modify your text to include the missing information. If you are certain that an item does not apply, please write "n/a" and provide a short explanation.

Upload your completed checklist as an extra file when you submit to a journal.

In your methods section, say that you used the STROBE cohortreporting guidelines, and cite them as:

von Elm E, Altman DG, Egger M, Pocock SJ, Gotzsche PC, Vandenbroucke JP. The Strengthening the Reporting of Observational Studies in Epidemiology (STROBE) Statement: guidelines for reporting observational studies.

For peer review only

		Reporting Item	Page Number
**Title and abstract**			
Title	#1a	Indicate the study's design with a commonly used term in the title or the abstract	1
Abstract	#1b	Provide in the abstract an informative and balanced summary of what was done and what was found	2
**Introduction**			
Background / rationale	#2	Explain the scientific background and rationale for the investigation being reported	4
Objectives	#3	State specific objectives, including any prespecified hypotheses	5
**Methods**			
Study design	#4	Present key elements of study design early in the paper	5
Setting	#5	Describe the setting, locations, and relevant dates, including periods	5

		of recruitment, exposure, follow-up, and data collection	
Eligibility criteria	#6a	Give the eligibility criteria, and the sources and methods of selection of participants. Describe methods of follow-up.	5
Eligibility criteria	#6b	For matched studies, give matching criteria and number of exposed and unexposed	6
Variables	#7	Clearly define all outcomes, exposures, predictors, potential confounders, and effect modifiers. Give diagnostic criteria, if applicable	7
Data sources / measurement	#8	For each variable of interest give sources of data and details of methods of assessment (measurement). Describe comparability of assessment methods if there is more than one group. Give information separately for for exposed and unexposed groups if applicable.	6
Bias	#9	Describe any efforts to address potential sources of bias	10
Study size	#10	Explain how the study size was arrived at	5
Quantitative variables	#11	Explain how quantitative variables were handled in the analyses. If applicable, describe which groupings were chosen, and why	6
Statistical methods	#12a	Describe all statistical methods, including those used to control for confounding	
7			
Statistical methods	#12b	Describe any methods used to examine subgroups and interactions	7
Statistical methods	#12c	Explain how missing data were addressed	7
Statistical methods	#12d	If applicable, explain how loss to follow-up was addressed	7
Statistical methods	#12e	Describe any sensitivity analyses	
7			
**Results**			
Participants	#13a	Report numbers of individuals at each stage of study—eg numbers potentially eligible, examined for eligibility, confirmed eligible,	5

For peer review only

		included in the study, completing follow-up, and analysed. Give information separately for for exposed and unexposed groups if applicable.	
Participants	#13b	Give reasons for non-participation at each stage	5
Participants	#13c	Consider use of a flow diagram	
5			
Descriptive data	#14a	Give characteristics of study participants (eg demographic, clinical, social) and information on exposures and potential confounders. Give information separately for exposed and unexposed groups if applicable.	7
Descriptive data	#14b	Indicate number of participants with missing data for each variable of interest	
7			
Descriptive data	#14c	Summarise follow-up time (eg, average and total amount)	
7			
Outcome data	#15	Report numbers of outcome events or summary measures over time. Give information separately for exposed and unexposed groups if applicable.	
7			
Main results	#16a	Give unadjusted estimates and, if applicable, confounder-adjusted estimates and their precision (eg, 95% confidence interval). Make clear which confounders were adjusted for and why they were included	9
Main results	#16b	Report category boundaries when continuous variables were categorized	7
Main results	#16c	If relevant, consider translating estimates of relative risk into absolute risk for a meaningful time period	
8			
Other analyses	#17	Report other analyses done—eg analyses of subgroups and interactions, and sensitivity analyses	9

Discussion

For peer review only

Key results	#18	Summarise key results with reference to study objectives	10
Limitations	#19	Discuss limitations of the study, taking into account sources of potential bias or imprecision. Discuss both direction and magnitude of any potential bias.	11
Interpretation	#20	Give a cautious overall interpretation considering objectives, limitations, multiplicity of analyses, results from similar studies, and other relevant evidence.	12
Generalisability	#21	Discuss the generalisability (external validity) of the study results	12
**Other Information**			
Funding	#22	Give the source of funding and the role of the funders for the present study and, if applicable, for the original study on which the present article is based	13

The STROBE checklist is distributed under the terms of the Creative Commons Attribution License CC-BY. This checklist was completed on 19. February 2023 using https://www.goodreports.org/, a tool made by the EQUATOR Network in collaboration with Penelope.ai

For peer review only

BMJ Open

Concurrent trajectories of part-time work and sickness absence - a longitudinal cohort study over 11 years among shift working hospital employees

Journal:	*BMJ Open*
Manuscript ID	bmjopen-2023-072987.R2
Article Type:	Original research
Date Submitted by the Author:	06-Sep-2023
Complete List of Authors:	Ropponen, Annina; Finnish Institute of Occupational Health; Karolinska Institute, Department of Clinical Neuroscience<br>Ervasti, Jenni; Finnish Institute of Occupational Health, Topeliuksenkatu 41 a A, FI-00250, Helsinki, Finland<br>Harma, Mikko; Finnish Institute of Occupational Health, Work Ability and Work Careers
**Primary Subject Heading**:	Epidemiology
Secondary Subject Heading:	Occupational and environmental medicine
Keywords:	EPIDEMIOLOGY, Human resource management < HEALTH SERVICES ADMINISTRATION & MANAGEMENT, OCCUPATIONAL & INDUSTRIAL MEDICINE

SCHOLARONE™
Manuscripts

I, the Submitting Author has the right to grant and does grant on behalf of all authors of the Work (as defined in the below author licence), an exclusive licence and/or a non-exclusive licence for contributions from authors who are: i) UK Crown employees; ii) where BMJ has agreed a CC-BY licence shall apply, and/or iii) in accordance with the terms applicable for US Federal Government officers or employees acting as part of their official duties; on a worldwide, perpetual, irrevocable, royalty-free basis to BMJ Publishing Group Ltd ("BMJ") its licensees and where the relevant Journal is co-owned by BMJ to the co-owners of the Journal, to publish the Work in this journal and any other BMJ products and to exploit all rights, as set out in our licence.

The Submitting Author accepts and understands that any supply made under these terms is made by BMJ to the Submitting Author unless you are acting as an employee on behalf of your employer or a postgraduate student of an affiliated institution which is paying any applicable article publishing charge ("APC") for Open Access articles. Where the Submitting Author wishes to make the Work available on an Open Access basis (and intends to pay the relevant APC), the terms of reuse of such Open Access shall be governed by a Creative Commons licence – details of these licences and which Creative Commons licence will apply to this Work are set out in our licence referred to above.

Other than as permitted in any relevant BMJ Author's Self Archiving Policies, I confirm this Work has not been accepted for publication elsewhere, is not being considered for publication elsewhere and does not duplicate material already published. I confirm all authors consent to publication of this Work and authorise the granting of this licence.

For peer review only

Concurrent trajectories of part-time work and sickness absence - a longitudinal cohort study over 11 years among shift working hospital employees

Authors: Annina Ropponen[1,2], Jenni Ervasti[1], Mikko Härmä[1]

[1] Finnish Institute of Occupational Health, Helsinki, Finland

[2] Division of Insurance Medicine, Department of Clinical Neuroscience, Karolinska Institute, Stockholm, Sweden

Corresponding author: Annina Ropponen, Finnish Institute of Occupational Health, P.O. Box 18, 00032 Finnish Institute of Occupational Health, Finland, email. annina.ropponen@ttl.fi

Word count: 2895.

For peer review only

Abstract

Objectives: To investigate the concurrent changes in part-time work and sickness absence (SA) in healthcare. Another aim was to investigate the role of age and sex on different concurrent trajectory groups.

Design: Prospective cohort study.

Setting: Public hospital districts (n = 10) and cities (n = 11) in Finland.

Participants: Payroll-based objective working hour data of the healthcare sector in Finland for 28969 employees in 2008-2019 were utilized. The final sample included those working shifts with three consecutive years of data and without baseline (≥14 days) SA.

Primary outcomes: Part-time work (yes or no) and months of SA.

Measures: Group-based trajectory modelling to identify concurrent changes in part-time work, and months of SA while controlling the time-variant amount of night work and multinomial regression models for relative risk (RR) with 95% confidence intervals (CI) were used.

Results: Four-group trajectory model was the best solution: Group 1 (61.2%) with full-time work and no SA, Group 2 (16.9%) with slowly increasing probability of part-time work and low but mildly increasing SA, Group 3 (17.6%) with increasing part-time work and no SA, and Group 4 (4.3%) with fluctuating, increasing part-time work and highest and increasing levels of SA. Men had a lower (RR 0.49-0.75), and older age groups had a higher likelihood (RRs 1.32-3.79) of belonging to trajectory groups 2-4.

Conclusions Most of the sample were in the trajectory group with full-time work and no SA. The Probability of part-time work increased over time, linked with concurrent low increase or no SA. A minor group of employees had both an increased probability of part-time work and SA. Part-time work and other solutions might merit attention to promote sustainable working life among healthcare employees.

For peer review only

Article summary

Strengths and limitations of this study

- Payroll-based register data of working hours enabled objective assessment of part-time work and sickness absence.
- A homogenous sample of hospital employees working in specialized healthcare services or at primary healthcare, including e.g., health centers, and local hospitals for elderly and chronically ill people providing 24/7 care, or home care for the disabled.
- Group-based trajectory model (GBTM) to identify concurrent trajectories of part-time work and mean number of sickness absence months/years for the follow-up from 2009 to 2019 to estimate a distinct pattern over time.
- Lack of information on the reasons for part-time work and no information on diagnosis or part-time sickness absence.

Keywords: shift work, part-time work, hospital, sick leave, longitudinal study

For peer review only

Background

The healthcare sector is facing major challenges due to population aging, increased need for care, and economic challenges combined with staff shortages. The aging population affects both ensuring sufficient levels of nursing staff, but also the growing demands for the care of the aging population [1, 2]. The existing need for longer work careers [3] combined with increasing turnover rates emphasizes the urgent need to understand working hours in association with work capacity and sustainable working life. This calls for an understanding of associations between working hours and work capacity and builds on sustainable work which is "working and living conditions that are such that they support people in engaging and remaining in work throughout an extended working life" [4]. For example, in recent years sickness absences (around 10% of all employed) have been increasing in Finland[5] and especially in healthcare [6]. In shift work, two working hour solutions may provide a possibility to decrease workload due to demanding working conditions or to arrange more time for care for small children or other dependents: an employee may work part-time, or to change from night work to day-time work [7, 8]. However, part-time work may also be health-related, i.e., being based on part-time sickness absence or disability pension [9, 10]. Until now, studies on the role of part-time work concerning future long-time trajectories of sickness absence are lacking. As sickness absence increases costs both to the employers and the society, further understanding of the linkage with part-time work is needed.

Working part-time may, besides influencing income, have long-term consequences on career and pension [11]. In Finland, every fifth woman (13% of men) works part-time [12]. On the other hand, shorter working hours and increased time for recovery could lengthen the work careers [13]. Due to an emergent need to increase employment rates and promote sustainable working life among the working population [14, 15], longitudinal studies investigating the role of part-time work are important. Many studies have reported associations between individual sociodemographic, socioeconomic, health- and work-related factors including part-time work for labor market exit due

For peer review only

to sickness absence or disability pension [16, 17]. However, until now, studies with longitudinal designs to investigate associations between part-time work and sustainable working life have mainly been based on survey data [15, 18-23].

This study hypothesized that due to aging, older employees may choose part-time work due to decreased health (reflected in higher sickness absence) more often than younger employees. We also hypothesize that part-time work without increased sickness absence might be more frequent among younger employees than older ones e.g., due to the need to combine work and life while having small children or with studies. The possibility to combine part-time work with part-time sickness absence, that is, to shorten working hours when having health problems and while compensated by social benefits (i.e., sickness absence benefits), may extend and/or maintain the working lives of the older employees [24]. However, part-time work and sickness absence may also both increase with age. Another aspect related to age is night work, i.e., older employees may wish to work more during the day than at night [25]. Also, age-related trends in the associations of night work and health have been identified [26, 27]. Therefore, based on the assumption that younger and older employees might react differently to night work [28, 29], one should control night work when investigating age-related differences in part-time work and sickness absence. This is relevant, especially in longitudinal settings in which working during the night may decrease due to the possibility of selecting more favorable working hours [30, 31].

This study aimed to investigate the concurrent changes in part-time work and sickness absence among employees in healthcare. Another aim was to investigate the role of age and sex on different concurrent trajectory groups.

Methods

This study was based on the ongoing Working Hours in the Finnish Public Sector study (WHFPS) [32, 33] with payroll-based register data of working hours for 2008-2019. To ensure follow-up across years and to exclude those with exceptionally long absences (due to sickness, parental leave, or

For peer review only

other reasons) and very short working periods, we restricted the sample to those with ≥31 work shifts/year in three consecutive years during 2008-2019. Then the sample was restricted to those without sickness absence (≥14 consequent days) at baseline in 2008. Last, only those with shift work contracts were included (Figure 1). The final sample was 28969 shift working employees in 10 hospital districts and 11 cities using Titania® shift scheduling software. In Finland, the hospital districts (45% of the final sample) are responsible for specialized healthcare services providing both specialized outpatient- and inpatient care. The healthcare employees in the cities (55% of the final sample) are those working at primary healthcare, including e.g., health centers, and local hospitals for elderly and chronically ill people providing 24/7 care, or home care for the disabled [33].

In this study, we utilized annual working hours and sickness absence data from 2008-2019 as has been described in detail before [32, 33]. Part-time work was based on work contract information and coded yes or no for each year based on the annual median. Then annual sickness absence of any length was measured in months/year for follow-up from 2009 to 2019. For the baseline 2008, the short sickness absences (≤3 days) were estimated. We also utilized the proportion of night work of all work shifts/year to estimate the time-varying effect of night work as a proxy of shift work in this study. Since aging employees may change from night work to day work [25, 34, 35], the proportion of night work was assumed to reflect this change, which in turn might affect the rate of part-time work. Baseline age (in 2008 and years categorized into < 25 years of age, ≥25 and <40 years, ≥40 and <55 years, and >55 years based on distribution), and sex were used in the analyses.

Patient and public involvement

Patients or the public were not involved in the design, or conduct, or reporting, or dissemination plans of our research.

Statistical analyses

First, we calculated the descriptive characteristics of the study sample. Then using a group-based multi-trajectory model, we identified concurrent trajectories of part-time work and mean number of

For peer review only

sickness absence months/years for the follow-up from 2009 to 2019 while accounting for night work as time varying covariate [36]. The group-based multi-trajectory method enables to identification of groups of individuals (trajectory groups) that follow a distinct pattern over time. The linear polynomial model was applied. Bayesian information criterion (BIC), Akaike Information Criteria (AIC), and average posterior probability (APP) were used to determine the best-fitting model. Third, we ran multinomial regression models for relative risk (RR) and 95% Confidence Intervals (CI) using sex and age groups to predict the group membership for each trajectory group. All statistical analyses were conducted with Stata 17.1 MP.

Results

At baseline in 2008, 7% of the final sample of 28969 shift working employees worked part-time, the mean age was 39.8 years (SD 10.7), 92% were women, and 34% had had at least one short sickness absence (≤3 days). The amount of night shifts was 8% of all shifts. During the follow-up (from 2009 to 2019), the mean number of sickness absence months varied from 0.3 months in 2009 to 0.6 months in 2018 (range 0-12 months).

A four-cluster model was chosen based on BIC and AIC values and parsimony for concurrent changes in part-time work and months of sickness absence (Table 1).

Table 1 Goodness of fit statistics of group-based trajectory analysis models

	Smallest group				
	N	%	BIC	AIC	APP
2-cluster model	4204	16.5	-190414.9	-190352.8	0.90
3-cluster model	3795	14.4	-181308.3	-181213.1	0.89
**4-cluster model***	**1099**	**4.3**	**-179517.1**	**-179388.9**	**0.87**
5-cluster model	1156	4.4	-175817.0	-175655.6	0.86
6-cluster model	1134	4.4	-175451.2	-175256.7	0.86
7-cluster model	688	3.7	-17117.5	-173890.0	0.79

* The models presented are shown in bold. BIC = Bayesian Information Criterion, AIC = Akaike Information Criterion, and APP = average posterior probability.

The concurrent trajectories were (Figure 2):

1. Group 1 (61.2% of the final sample) with full-time work and no sickness absence.

For peer review only

2. Group 2 (16.9) with slowly increasing probability of part-time work and low but mildly increasing sickness absence.
3. Group 3 (17.6%) with increasing part-time work and no sickness absence.
4. Group 4 (4.3%) with fluctuating but increasing part-time work and highest and increasing levels of sickness absence.

The regression models (Table 2) showed that compared to the youngest age group (<25 years of age at the baseline), the older age groups had an increased likelihood of belonging to trajectory groups 2-4 (in comparison to trajectory group 1). Men had a lower likelihood of belonging to trajectory groups 2-4 than women (Table 2).

Table 2 Sex and age group associated with cluster membership (part-time work and months of sickness absence). Multinomial regression (ref=Cluster 1 'Full-time work and no sickness absence', n=2124). Relative risk ratio (RR) with 95% confidence intervals (CI)

	**Group 2** Slowly increasing the probability of part-time work and low but mildly increasing sickness absence		**Group 3** Increasing part-time work and no sickness absence		**Group 4** Fluctuating but increasing part-time work and highest and increasing levels of sickness absence	
	**RR***	**95%CI**	**RR**	**95%CI**	**RR**	**95%CI**
Women	1		1		1	
Men	**0.74**	**0.63, 0.86**	**0.75**	**0.65, 0.87**	**0.49**	**0.34, 0.71**
Age <25 years	1		1		1	
25-39 years	**1.32**	**1.13, 1.54**	**1.56**	**1.36, 1.80**	**2.36**	**1.56, 3.56**
40-54 years	**1.83**	**1.58, 2.12**	1.10	0.95, 1.26	**3.79**	**2.54, 5.66**
≥55 years	**1.69**	**1.39, 2.07**	**1.53**	**1.27, 1.85**	**2.99**	**1.83, 4.88**

* Statistically significant RR with 95%CI in boldface

In the supplemental material, we present the concurrent trajectories without adjusting for time-variant night work (Supplemental Figure 1), and for each age group separately (Supplemental Figures S2-5) and their fit indexes in the supplemental tables S1-2. Although trajectory groups 1-3 are similar to adjusting for time-variant night work, group 4 indicates some changes in the probability of part-time work. Except for those aged 25-39 years with four trajectory groups, all other age groups were

For peer review only

best fitted to a five-group trajectory solution. The concurrent changes in part-time work and sickness absence varied between age groups: among the oldest age group, ≥55 years, three out of four trajectory groups had an increase in part-time work associated with low or no sickness absence. The concurrent trajectories with the decrease in part-time work (transition to full-time) with no sickness absence were identified among middle age groups (25-54 years).

Discussion

This longitudinal study of nearly 29 000 Finnish employees in the healthcare sector investigated the concurrent changes in part-time work and sickness absence across 11 years, and the role of age and sex on the identified concurrent trajectories. Four trajectory groups were detected: Group 1 (61%) with full-time work and no sickness absence, Group 2 (17%) with slowly increasing probability of part-time work and low but mildly increasing sickness absence, Group 3 (18%) with increasing part-time work and no sickness absence, and Group 4 (4%) with fluctuating but increasing part-time work and highest and increasing levels of sickness absence. Women and older employees were more likely to belong to groups 2-4 characterized by increasing part-time work. This is among the first studies to investigate the concurrent changes in part-time work and sickness absence from 2009 to 2019 while controlling for night work among healthcare employees. Hence, this study adds to the earlier knowledge based on survey data [15, 18-23].

The largest trajectory group (61%) worked full-time and had no sickness absence reflecting the well-known trend of women working mostly full-time in the Nordic countries [11]. However, we also detected two moderate size groups (trajectory groups 2 and 3) with increasing probability of part-time work and low but mildly increasing or no sickness absence. These might be indicative of our hypotheses that a) older employees may choose part-time work due to decreasing health (with higher sickness absence), or b) part-time work with low or no sickness absence might appear among younger employees due to the need to balance work and home life. Since the fourth trajectory group, although small (4%), indicated fluctuating, but increasing part-time work and highest and

For peer review only

increasing sickness absence, it seems to support the above-mentioned hypothesis, and the assumption that both part-time work and sickness absence increase along with age. Our Supplemental Figure S1 confirmed that overall, the four group trajectory solution was the best even without time variant night work. However, when night work was not accounted for (Supplemental Figure S1), the probability of part-time work was less likely indicating that transitioning to day work may be another strategy to affect the working hours along age [25]. This might be an important notion for workplaces and occupational healthcare since age-related trends in the associations of night work and health exist [26, 27] and perhaps modifications to working hours could promote sustainable working life [14, 15] if part-time work would be compensated with social benefits.

We were specifically interested in the role of sex and age on these concurrent changes of part-time work and sickness absence. Women had a higher likelihood of belonging to trajectory groups 2-4 characterized by increases in part-time work and with or without sickness absence. This is in line with earlier findings indicating that e.g., a typical employee having long-term sickness absence is a woman in her 50's and working in the healthcare sector [37, 38]. This might be because women have higher sickness absence rates than men [39-41], but also because women outnumber men in the healthcare sector (in our study, only 8% were men). Another influential, well-known fact is that men and women work part-time due to different reasons such as child care or health [11] and even societal factors such as the availability of daycare for children or salary levels can play a role [42-44]. Older age groups had a higher likelihood of belonging to trajectory groups 2-4 compared to group 1. There are some possible explanations. First, the concurrent increase of part-time work and full-time sickness absence along with age. Alternatively age-related trends of reacting to night work [28, 29], i.e., across age groups the concurrent changes of part-time work and sickness absence were different as shown in our supplement figures. Thus, further studies would be needed to address the various reasons for part-time work (e.g., health, availability of work), health issues (diagnoses for sickness absence), but also more detailed periods (e.g., monthly, or weekly), and working hour-based measures of part-time work. In addition, non-observational research designs are also needed.

For peer review only

We had access to objective working hour data including sickness absence from 2008 to 2019 almost in total 240000 Finnish employees in the healthcare sector. This unique dataset enabled us to restrict the sample to those working shift work, having complete data for ≤3 consecutive years during 2009-2019, and without baseline ≥14 days of sickness absence. These are evident strengths although the final sample size was around 29000 employees. Such data is free from reporting or memory bias adding to earlier knowledge based on mainly survey data [15, 18-23]. However, a limitation is that we had no information on the reasons for part-time work which may be due to e.g., health, personal matters, or unavailability of full-time work [45, 46]. We also cannot rule out the healthy worker bias but assume it might not play a major role. Furthermore, we only had information on sickness absence without diagnosis and no information on part-time sickness absence. Since part-time work may relate to health status, i.e., being based on part-time sickness absence or disability pension [9, 10], detailed health information should be accounted for in further studies. We also lacked information on many sociodemographic factors (i.e., marital status, number of children, level of income) which is a limitation. Further studies should be designed to address these factors. As in any observational study, we were unable to demonstrate the causality between part-time work and sickness absence. It may be that transfer into part-time work indicates health problems and thus is a marker or a risk factor for subsequent sickness absence. However, part-time work may also be a stepping stone when returning to work after sickness absence. Future research on the interrelations between these two phenomena is needed. Yet another limitation might be the generalizability of the findings. In Finland, and Nordic countries, work in healthcare is organized mostly via irregular working hours, i.e., shift work with a non-standard working hour schedule with variation in start and finish times, lengths, and rest periods between work shifts [47]. On the other hand, sickness absence is subsidized via the welfare society model typical to Nordic countries. Hence our findings might be more generalizable to Nordic than other countries.

Conclusions

For peer review only

The majority (61%) of employees worked full-time and without sickness absence throughout the 11-year follow-up. However, for the rest of the employees, the probability of part-time work increased over time with varying levels of simultaneous changes in sickness absence. An increase in part-time work was more probable for women and older employees. For a minority of the employees (4%), the increase in part-time work was strongly linked with the concurrent increase in sickness absence. The relationship between part-time work and sickness absence is likely bidirectional. Transfer into part-time work may indicate health problems and subsequent sickness absence, but part-time work may also be used when gradually returning to work after a sickness absence and thus it can promote sustainable working life in healthcare employees.

For peer review only

Statements and declarations

Funding statement

This work was supported by the Academy of Finland, DIGIHUM-programme grant number 329200.

Conflict of interest

The authors declare that they have no conflict of interest.

Author contributions

AR, MH, and JE contributed to the study's conception and design. Material preparation, data collection, and analysis were performed by AR. The first draft of the manuscript was written by AR, and all the authors commented on previous versions of the manuscript. All the authors read and approved the final manuscript.

Availability of data and materials

The data that support the findings of this study are available from the Hospital districts and Cities but restrictions apply to the availability of these data, which were used under license for the current study, and so are not publicly available. Data are however available from the last author upon reasonable request and with permission of the Hospital districts and Cities.

Ethics approval

This study was fully based on employer-owned administrative employment register data that the Hospital Districts and Cities had permitted access to and applied pseudonymized identification numbers for research purposes. Using such data does not include any experimental protocols requiring approval. In Finland, research using such data does not need to undergo review by an ethics committee according to national legislation (Medical Research Act).

Consent to participate

For peer review only

The consent to participate was not applicable since the data comprised employer-owned employment information and was analyzed as administrative register data pseudonymized for research purposes.

Consent for publication

Not applicable.

For peer review only

Figure legends

Figure 1 Sample selection

Figure 2 Four clusters of trajectories of part-time work and months of sickness absence adjusted for time-variant night work (95% CI are shown as dotted lines, but poorly visible due to being very narrow)

For peer review only

References

1. Tursunbayeva A. Human resource technology disruptions and their implications for human resources management in healthcare organizations. *BMC Health Serv Res* 2019;19(1):268. doi: 10.1186/s12913-019-4068-3 [published Online First: 2019/05/01]
2. Smiley RA, Lauer P, Bienemy C, et al. The 2017 National Nursing Workforce Survey. *Journal of Nursing Regulation* 2018;9(3):S4-S88. doi: Doi 10.1016/S2155-8256(18)30131-5
3. Vanroelen C. Longer working careers and sustainable work: the issue of social inequality. *Society Health & Vulnerability* 2017;8(sup1):1332854. doi: Unsp 1332854

10.1080/20021518.2017.1332854
4. Eurofound. Sustainable working over the life course: concept paper. Publications Office of the European Union, Luxembourg, 2015.
5. Kela. Information package: Sickness absence 2023 [Available from: https://tietotarjotin.fi/en/information-package/2699253/information-package-sickness-absence accessed 1st of August 2023.
6. Health FIoO. Sickness absence in Finnish municipalities 2019 [Available from: https://www.tyoelamatieto.fi/en/dashboards/kunta10-sick-leave accessed 1st of August 2023.
7. Steinmetz S, de Vries DH, Tijdens KG. Should I stay or should I go? The impact of working time and wages on retention in the health workforce. *Hum Resour Health* 2014;12:23. doi: 10.1186/1478-4491-12-23 [published Online First: 2014/04/25]
8. Flodgren GM, Bidonde J. Effects and experiences of part-time work in the health- and community-care services: a scoping review. (Effekter og erfaringer av deltidsarbeid i helse- og omsorgstjenesten: en kartleggingsoversikt). Oslo: National Institute of Public Health, 2019.
9. Schram JL, Solovieva S, Leinonen T, et al. The influence of occupational class and physical workload on working life expectancy among older employees. *Scandinavian journal of work, environment & health* 2021;47(1):5-14. doi: 10.5271/sjweh.3919 [published Online First: 2020/09/01]
10. Ervasti J, Kausto J, Koskinen A, et al. Labor Market Participation Before and After Long-Term Part-Time Sickness Absence in Finland: A Population-Based Cohort Study. *Journal of Occupational and Environmental Medicine* 2020;62(4)
11. NIKK. Faktablad: Vem jobbar och när? Norway: Nordiska ministerrådet, 2018.
12. OECD. Part-time employment rate (indicator) 2023 [accessed 1st of August 2023.
13. Härmä MI, Ilmarinen JE. Towards the 24-hour society--new approaches for aging shift workers? *Scand J Work Environ Health* 1999;25(6):610-5. doi: 10.5271/sjweh.488 [published Online First: 2000/07/07]
14. Health MoSAa. Eheä yhteiskunta ja kestävä hyvinvointi Sosiaali- ja terveysministeriön tulevaisuuskatsaus. Valtioneuvoston julkaisusarja 22/2018. Helsinki: Sosiaali- ja terveysministeriö, 2018.
15. de Wind A, van der Pas S, Blatter BM, et al. A life course perspective on working beyond retirement-results from a longitudinal study in the Netherlands. *BMC Public Health* 2016;16:499. doi: 10.1186/s12889-016-3174-y [published Online First: 2016/06/12]
16. Reinholdt S, Upmark M, Alexanderson K. Health-selection mechanisms in the pathway towards a disability pension. *Work* 2010;37(1):41-51. doi: 10.3233/WOR-2010-1055 [published Online First: 2010/09/23]
17. Ropponen A, Alexanderson K, Svedberg P. Part-time work or social benefits as predictors for disability pension: a prospective study of Swedish twins. *Int J Behav Med* 2014;21(2):329-36. doi: 10.1007/s12529-013-9303-4 [published Online First: 2013/03/13]
18. Lu W, Benson R, Glaser K, et al. Relationship between employment histories and frailty trajectories in later life: evidence from the English Longitudinal Study of Ageing. *J Epidemiol*

For peer review only

Community Health 2017;71(5):439-45. doi: 10.1136/jech-2016-207887 [published Online First: 2016/12/04]
19. Sohier L, Van Ootegem L, Verhofstadt E. Well-Being During the Transition from Work to Retirement. *Journal of Happiness Studies* 2021;22(1):263-86. doi: 10.1007/s10902-020-00228-6
20. Komp-Leukkunen K. Life-histories in Finland: First results from the Survey of Health, Ageing and Retirement in Europe Working papers. Helsinki: Population Research Institute, 2021.
21. Ek E, Ala-Mursula L, Velázquez RG, et al. Employment trajectories until midlife associate with early social role investments and current work-related well-being. *Advances in Life Course Research* 2021;47:100391. doi: https://doi.org/10.1016/j.alcr.2020.100391
22. McDonough P, Worts D, Corna LM, et al. Later-life employment trajectories and health. *Advances in Life Course Research* 2017;34:22-33. doi: https://doi.org/10.1016/j.alcr.2017.09.002
23. de Wind A, Scharn M, Geuskens GA, et al. Predictors of working beyond retirement in older workers with and without a chronic disease - results from data linkage of Dutch questionnaire and registry data. *BMC Public Health* 2018;18(1):265. doi: 10.1186/s12889-018-5151-0 [published Online First: 2018/02/20]
24. Kausto J, Viikari-Juntura E, Virta LJ, et al. Effectiveness of new legislation on partial sickness benefit on work participation: a quasi-experiment in Finland. *BMJ Open* 2014;4(12):e006685. doi: 10.1136/bmjopen-2014-006685
25. Härmä M. Workhours in relation to work stress, recovery and health. *Scand J Work Environ Health* 2006;32(6):502-14.
26. Booker LA, Magee M, Rajaratnam SMW, et al. Individual vulnerability to insomnia, excessive sleepiness and shift work disorder amongst healthcare shift workers. A systematic review. *Sleep Med Rev* 2018;41:220-33. doi: 10.1016/j.smrv.2018.03.005 [published Online First: 2018/04/24]
27. Ritonja J, Aronson KJ, Matthews RW, et al. Working Time Society consensus statements: Individual differences in shift work tolerance and recommendations for research and practice. *Ind Health* 2019;57(2):201-12. doi: 10.2486/indhealth.SW-5 [published Online First: 2019/02/01]
28. Cai C, Vandermeer B, Khurana R, et al. The impact of occupational shift work and working hours during pregnancy on health outcomes: a systematic review and meta-analysis. *Am J Obstet Gynecol* 2019;221(6):563-76. doi: 10.1016/j.ajog.2019.06.051 [published Online First: 2019/07/06]
29. Stock D, Knight JA, Raboud J, et al. Rotating night shift work and menopausal age. *Hum Reprod* 2019;34(3):539-48. doi: 10.1093/humrep/dey390 [published Online First: 2019/02/13]
30. Hess M, Bauknecht J, Pink S. Working Hours Flexibility and Timing of Retirement: Findings from Europe. *J Aging Soc Policy* 2018;30(5):478-94. doi: 10.1080/08959420.2018.1500857 [published Online First: 2018/08/02]
31. Vanajan A, Bultmann U, Henkens K. Health-related Work Limitations Among Older Workers-the Role of Flexible Work Arrangements and Organizational Climate. *The Gerontologist* 2019 doi: 10.1093/geront/gnz073 [published Online First: 2019/06/01]
32. Harma M, Ropponen A, Hakola T, et al. Developing register-based measures for assessment of working time patterns for epidemiologic studies. *Scand J Work Environ Health* 2015;41(3):268-79. doi: 10.5271/sjweh.3492 [published Online First: 2015/03/20]
33. Härmä MI, Shiri R, Ervasti J, et al. National Recommendations for Shift Scheduling in Healthcare: A 5-Year Prospective Cohort Study on Working Hour Characteristics. 2022 doi: http://dx.doi.org/10.2139/ssrn.4084020
34. Blok MM, de Looze MP. What is the evidence for less shift work tolerance in older workers? *Ergonomics* 2011;54(3):221-32. doi: 10.1080/00140139.2010.548876

For peer review only

35. Härmä M, Karhula K, editors. *Working hours, health, well-being and participation in working life : Current knowledge and recommendations for health and safety*. Helsinki, Finland: Työterveyslaitos, 2020.
36. Nagin DS, Jones BL, Passos VL, et al. Group-based multi-trajectory modeling. *Stat Methods Med Res* 2018;27(7):2015-23. doi: 10.1177/0962280216673085 [published Online First: 2018/05/31]
37. Försäkrings A. Psykisk ohälsa i olika län, 2019.
38. samhällsmedicin Cfeo. Coronapandemin och psykisk hälsa. En prognos om kort- och långsiktiga effekter. Stockholm: Region Stockholm, 2020.
39. Borg K, Hensing G, Alexanderson K. Risk factors for disability pension over 11 years in a cohort of young persons initially sick-listed with low back, neck, or shoulder diagnoses. *Scand J Public Health* 2004;32(4):272-8. doi: 10.1080/14034940310019524
40. Haukenes I, Gjesdal S, Rortveit G, et al. Women's higher likelihood of disability pension: the role of health, family and work. A 5-7 years follow-up of the Hordaland Health Study. *BMC public health* 2012;12:720. doi: 10.1186/1471-2458-12-720
41. Mykletun A, Overland S, Dahl AA, et al. A population-based cohort study of the effect of common mental disorders on disability pension awards. *Am J Psychiatry* 2006;163(8):1412-8. doi: 10.1176/ajp.2006.163.8.1412 [published Online First: 2006/08/01]
42. Barbieri P, Cutuli G, Guetto R, et al. Part-time employment as a way to increase women's employment: (Where) does it work? *International Journal of Comparative Sociology* 2019;60(4):249-68. doi: 10.1177/0020715219849463
43. EuroStat. Being young in Europe today - labour market - access and participation 2020 [Available from: https://ec.europa.eu/eurostat/statistics-explained/index.php?title=Being_young_in_Europe_today_-_labour_market_-_access_and_participation.
44. Viikari-Juntura E, Leinonen T, Virta LJ, et al. Early part-time sick leave results in considerable savings in social security costs at national level: an analysis based on a quasi-experiment in Finland. *Scand J Work Environ Health* 2019;45(2):203-08. doi: 10.5271/sjweh.3780 [published Online First: 2018/10/20]
45. Pech C, Klainot-Hess E, Norris D. Part-time by Gender, Not Choice: The Gender Gap in Involuntary Part-time Work. *Sociological Perspectives* 2021;64(2):280-300. doi: 10.1177/0731121420937746
46. Rui F, Jennifer G, Peter CC. *Research Square* 2021 doi: 10.21203/rs.3.rs-390046/v1
47. Sallinen M, Kecklund G. Shift work, sleep, and sleepiness - differences between shift schedules and systems. *Scand J Work Environ Health* 2010;36(2):121-33. doi: 10.5271/sjweh.2900 [published Online First: 2010/02/02]

For peer review only

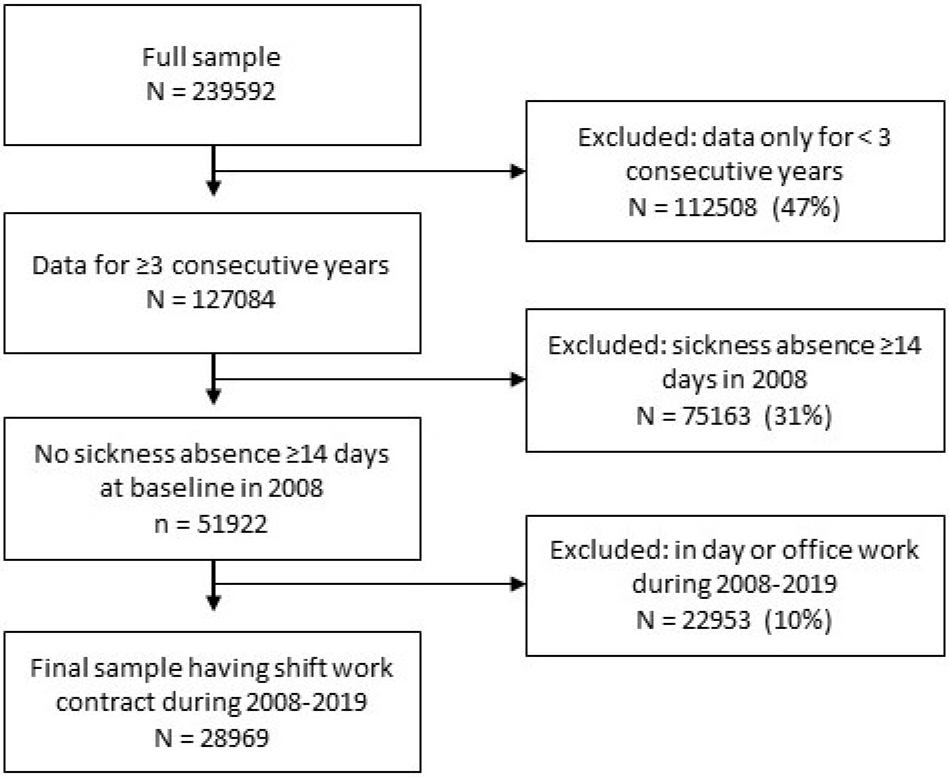

127x104mm (96 x 96 DPI)

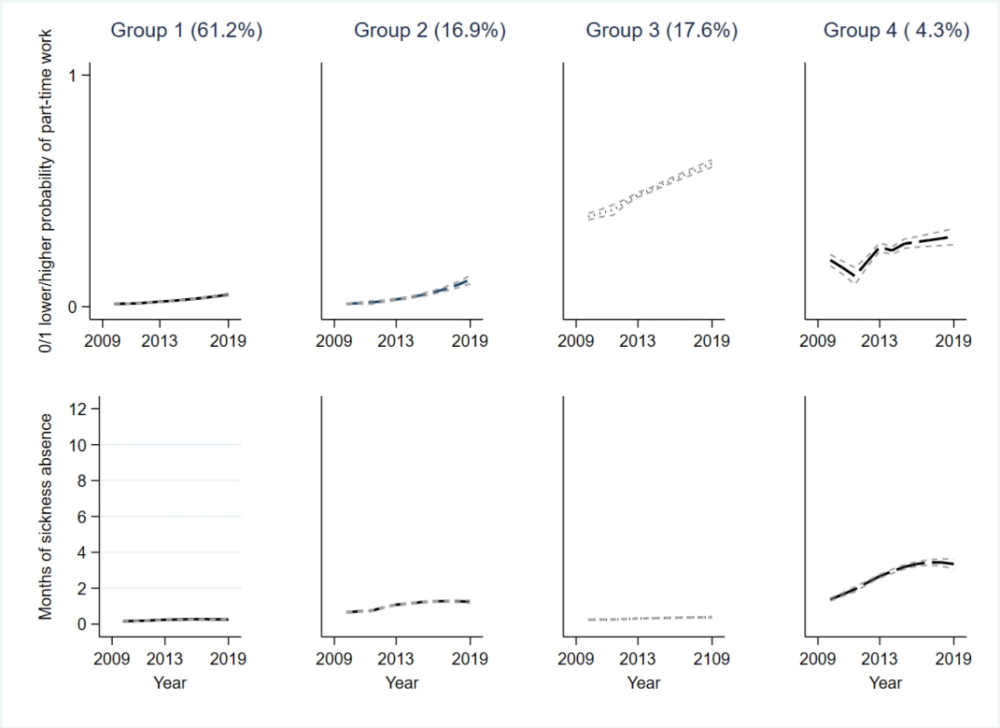

428x312mm (72 x 72 DPI)

Supplemental material

Concurrent trajectories of part-time work and sickness absence - a longitudinal cohort study over 11 years among shift working hospital employees

For peer review only

Supplemental material

Supplemental Table S1 Goodness of fit statistics of group-based trajectory analysis models

	Smallest group				
	**N**	**%**	**BIC**	**AIC**	**APP**
2-cluster model	5461	17	-263123.4	-263077.8	0.92
3-cluster model	5184	16	-254453.0	-254382.6	0.91
**4-cluster model***	**1671**	**5**	**-250141.8**	**-250046.5**	**0.91**
5-cluster model	919	4	-248427.7	-248307.6	0.84
6-cluster model	920	4	-244756.5	-244611.4	0.83

* The models presented are shown in bold. BIC = Bayesian Information Criterion, AIC = Akaike Information Criterion, and APP = average posterior probability.

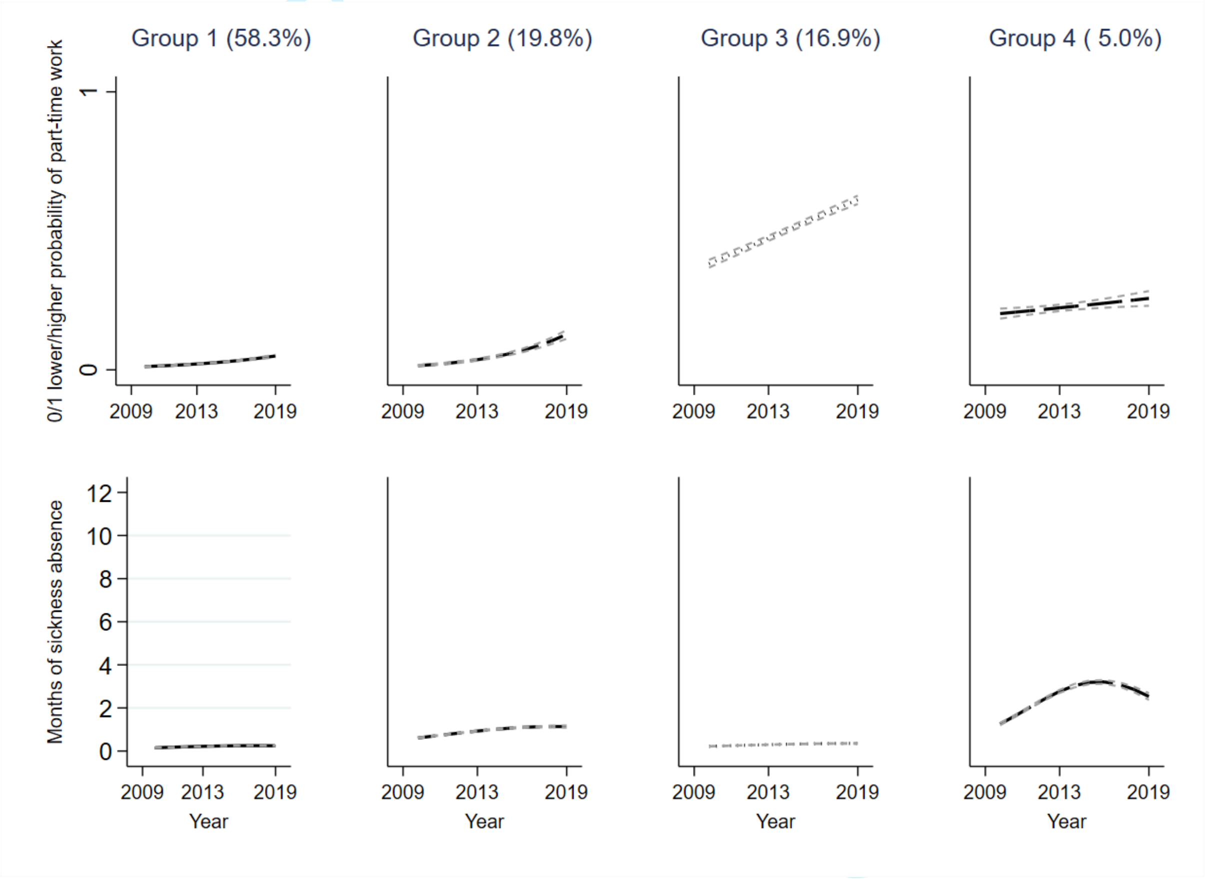

Supplemental Figure S1 Four clusters of trajectories of part-time work and months of sickness absence (95% CI are shown as dotted lines, but poorly visible due to being very narrow)

Supplemental material

Supplemental Table S2 Goodness of fit statistics of group-based trajectory analysis models

	Baseline age < 25 years (n = 1950)					Baseline age ≥25 and <40 years (n = 7437)				
	**Smallest group**					**Smallest group**				
	**N**	**%**	**BIC**	**AIC**	**APP**	**N**	**%**	**BIC**	**AIC**	**APP**
2-cluster model	453	25	-13732.04	-13690.23	0.90	1790	25	-63056.56	-63004.71	0.93
3-cluster model	235	14	-13234.66	-13170.54	0.88	928	13	-59538.30	-59458.79	0.90
**4-cluster model***	95	6	-13196.39	-13109.97	0.78	**649**	**10**	**-58972.56**	**-58865.39**	**0.86**
**5-cluster model***	**80**	**5**	**-13183.34**	**-13074.62**	**0.83**	274	4	-58496.44	-58361.61	0.88
6-cluster model	70	4	-13142.51	-13011.84	0.81					
7-cluster model	17	1	-13118.99	-12965.66	0.93					
	**Baseline age ≥40 and <55 years (n = 10143)**					**Baseline age ≥55 years (n = 1430)**				
2-cluster model	2626	26	-94282.84	-94228.65	0.94	322	23	-12128.61	-12089.12	0.97
3-cluster model	1120	11	-89502.96	-89419.88	0.94	259	19	-11463.96	-11403.41	0.91
4-cluster model	597	6	-87992.44	-87880.46	0.91	154	10	-11295.51	-11213.90	0.91
**5-cluster model***	**535**	**5**	**-87053.32**	**-86912.44**	**0.91**	**139**	**10**	**-11202.11**	**-11099.44**	**0.84**
6-cluster model	277	3	-86516.63	-86346.85	0.87	54	4	-11094.27	-10970.53	0.89

* The models presented are shown in bold. BIC = Bayesian Information Criterion, AIC = Akaike Information Criterion, and APP = average posterior probability.

For peer review only

Supplemental material

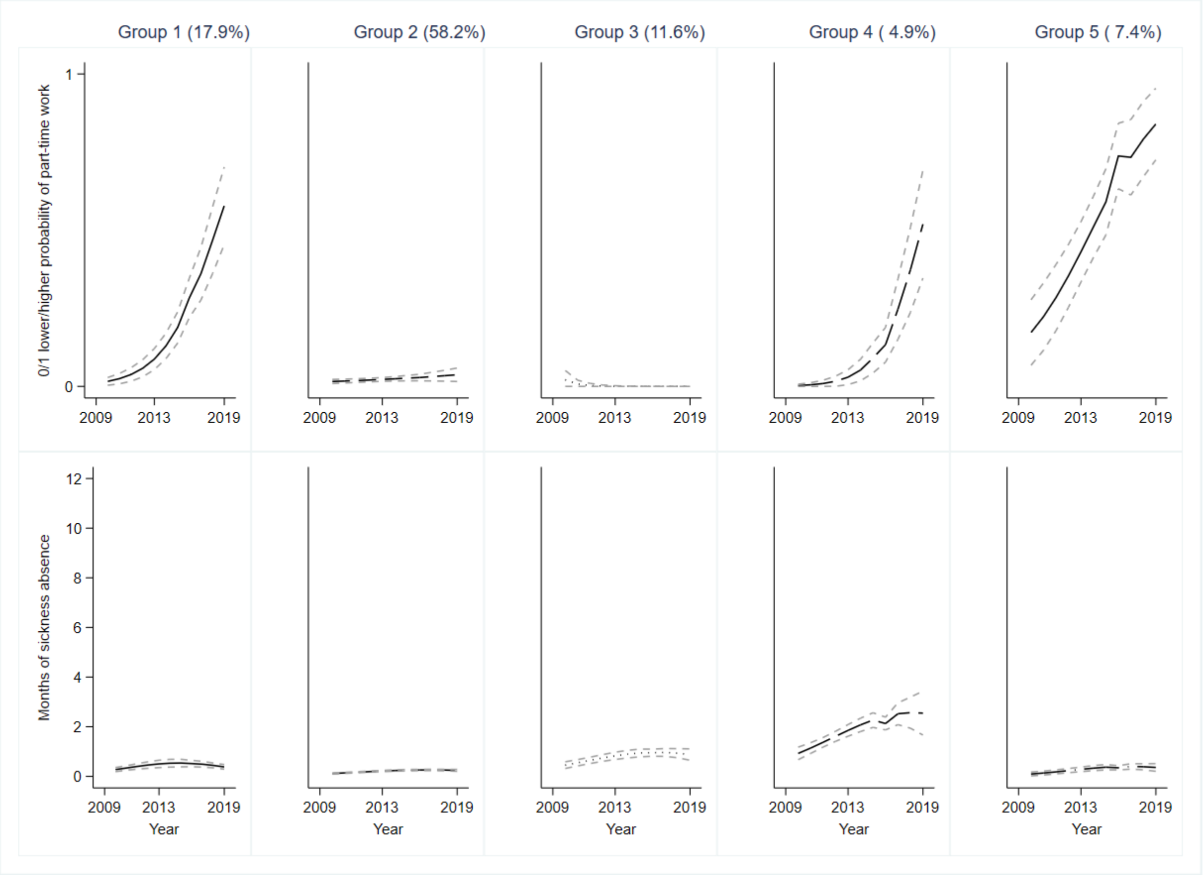

Supplemental Figure S2 Five clusters of trajectories of part-time work and months of sickness absence **among those with baseline age <25 years** (n = 1950) adjusted for time-variant night work (95% CI are shown as dotted lines, but poorly visible due to being very narrow)

Supplemental material

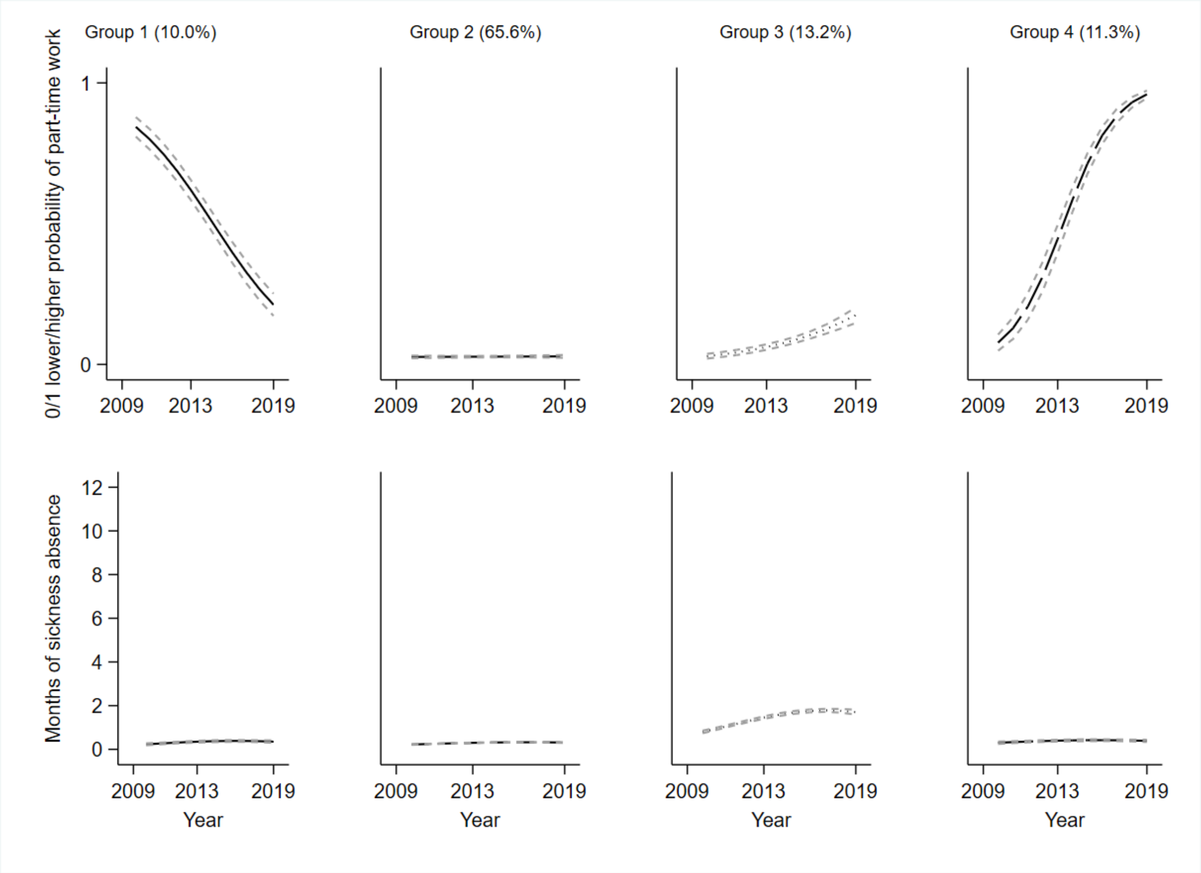

Supplemental Figure S3 Four clusters of trajectories of part-time work and months of sickness absence **among those with baseline age ≥25 and <40 years** (n = 7437) adjusted for time-variant night work (95% CI are shown as dotted lines, but poorly visible due to being very narrow)

Supplemental material

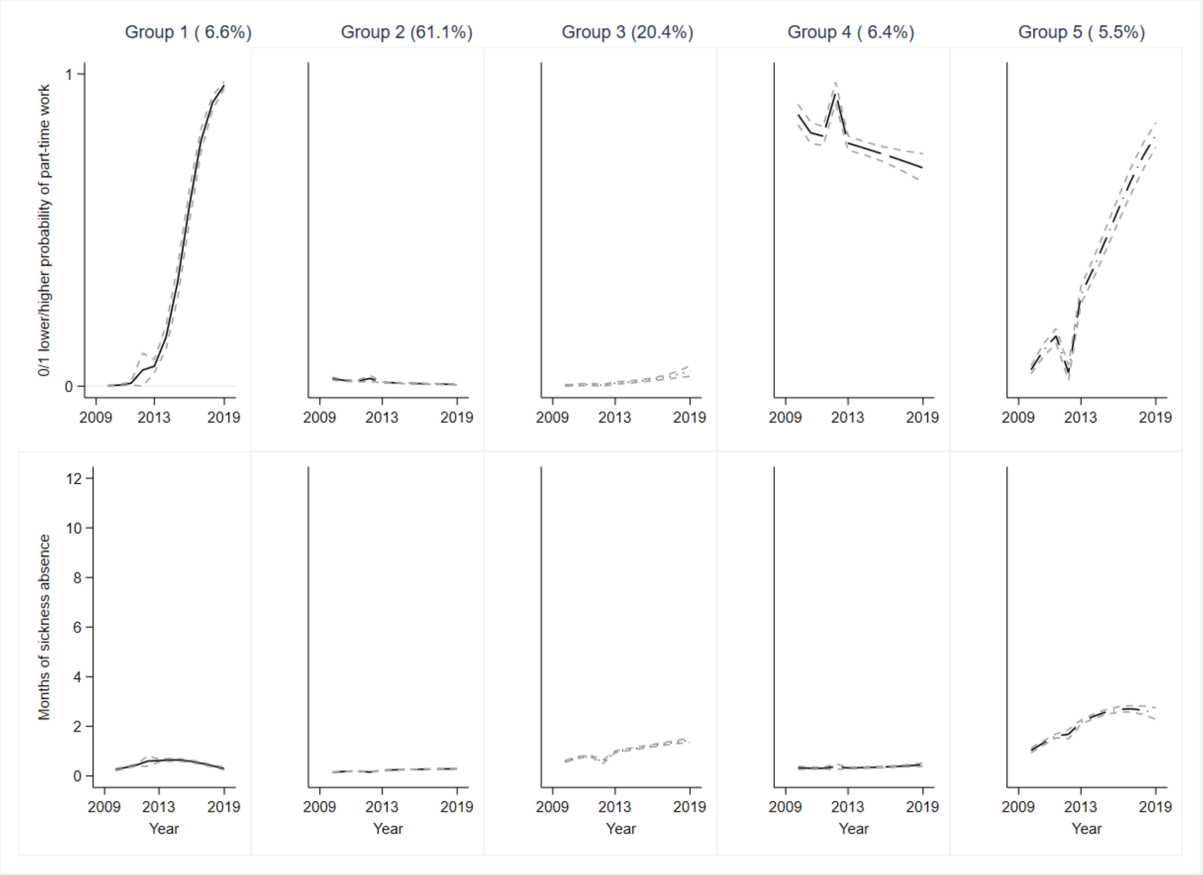

Supplemental Figure S4 Five clusters of trajectories of part-time work and months of sickness absence **among those with baseline age ≥40 and <55 years** (n = 10143) adjusted for time-variant night work (95% CI are shown as dotted lines, but poorly visible due to being very narrow)

Supplemental material

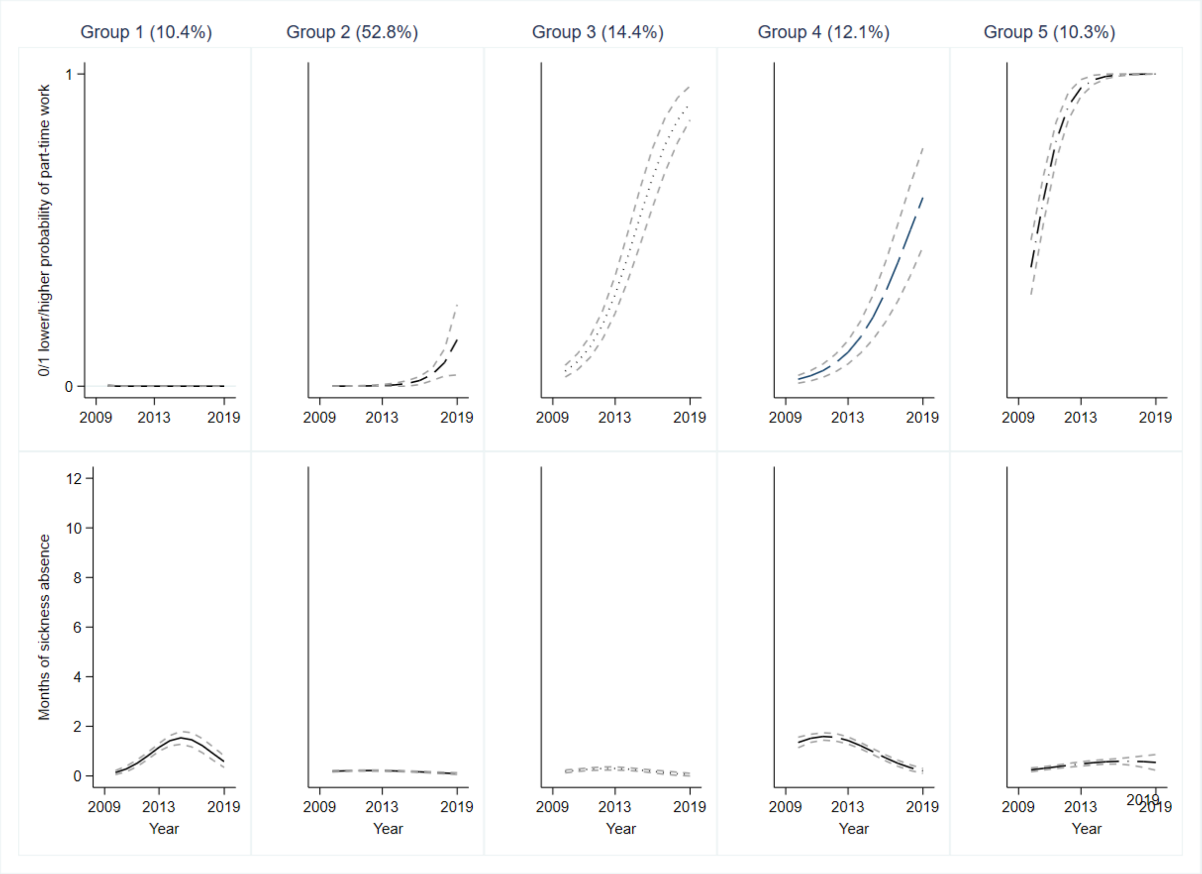

Supplemental Figure S5 Five clusters of trajectories of part-time work and months of sickness absence **among those with baseline age ≥55 years** (n = 1430) adjusted for time-variant night work (95% CI are shown as dotted lines, but poorly visible due to being very narrow)

review only

Reporting checklist for cohort study.

Based on the STROBE cohort guidelines.

Instructions to authors

Complete this checklist by entering the page numbers from your manuscript where readers will find each of the items listed below.

Your article may not currently address all the items on the checklist. Please modify your text to include the missing information. If you are certain that an item does not apply, please write "n/a" and provide a short explanation.

Upload your completed checklist as an extra file when you submit to a journal.

In your methods section, say that you used the STROBE cohortreporting guidelines, and cite them as:

von Elm E, Altman DG, Egger M, Pocock SJ, Gotzsche PC, Vandenbroucke JP. The Strengthening the Reporting of Observational Studies in Epidemiology (STROBE) Statement: guidelines for reporting observational studies.

For peer review only

		Reporting Item	Page Number
**Title and abstract**			
Title	#1a	Indicate the study’s design with a commonly used term in the title or the abstract	1
Abstract	#1b	Provide in the abstract an informative and balanced summary of what was done and what was found	2
**Introduction**			
Background / rationale	#2	Explain the scientific background and rationale for the investigation being reported	4
Objectives	#3	State specific objectives, including any prespecified hypotheses	5
**Methods**			
Study design	#4	Present key elements of study design early in the paper	5
Setting	#5	Describe the setting, locations, and relevant dates, including periods	5

		of recruitment, exposure, follow-up, and data collection	
Eligibility criteria	#6a	Give the eligibility criteria, and the sources and methods of selection of participants. Describe methods of follow-up.	5
Eligibility criteria	#6b	For matched studies, give matching criteria and number of exposed and unexposed	6
Variables	#7	Clearly define all outcomes, exposures, predictors, potential confounders, and effect modifiers. Give diagnostic criteria, if applicable	7
Data sources / measurement	#8	For each variable of interest give sources of data and details of methods of assessment (measurement). Describe comparability of assessment methods if there is more than one group. Give information separately for for exposed and unexposed groups if applicable.	6
Bias	#9	Describe any efforts to address potential sources of bias	10
Study size	#10	Explain how the study size was arrived at	5
Quantitative variables	#11	Explain how quantitative variables were handled in the analyses. If applicable, describe which groupings were chosen, and why	6
Statistical methods	#12a	Describe all statistical methods, including those used to control for confounding	
7			
Statistical methods	#12b	Describe any methods used to examine subgroups and interactions	7
Statistical methods	#12c	Explain how missing data were addressed	7
Statistical methods	#12d	If applicable, explain how loss to follow-up was addressed	7
Statistical methods	#12e	Describe any sensitivity analyses	
7			
**Results**			
Participants	#13a	Report numbers of individuals at each stage of study—eg numbers potentially eligible, examined for eligibility, confirmed eligible,	5

For peer review only

		included in the study, completing follow-up, and analysed. Give information separately for for exposed and unexposed groups if applicable.	
Participants	#13b	Give reasons for non-participation at each stage	5
Participants	#13c	Consider use of a flow diagram	
5			
Descriptive data	#14a	Give characteristics of study participants (eg demographic, clinical, social) and information on exposures and potential confounders. Give information separately for exposed and unexposed groups if applicable.	7
Descriptive data	#14b	Indicate number of participants with missing data for each variable of interest	
7			
Descriptive data	#14c	Summarise follow-up time (eg, average and total amount)	
7			
Outcome data	#15	Report numbers of outcome events or summary measures over time. Give information separately for exposed and unexposed groups if applicable.	
7			
Main results	#16a	Give unadjusted estimates and, if applicable, confounder-adjusted estimates and their precision (eg, 95% confidence interval). Make clear which confounders were adjusted for and why they were included	9
Main results	#16b	Report category boundaries when continuous variables were categorized	7
Main results	#16c	If relevant, consider translating estimates of relative risk into absolute risk for a meaningful time period	
8			
Other analyses	#17	Report other analyses done—eg analyses of subgroups and interactions, and sensitivity analyses	9

Discussion

For peer review only

Key results	#18	Summarise key results with reference to study objectives	10
Limitations	#19	Discuss limitations of the study, taking into account sources of potential bias or imprecision. Discuss both direction and magnitude of any potential bias.	11
Interpretation	#20	Give a cautious overall interpretation considering objectives, limitations, multiplicity of analyses, results from similar studies, and other relevant evidence.	12
Generalisability	#21	Discuss the generalisability (external validity) of the study results	12
**Other Information**			
Funding	#22	Give the source of funding and the role of the funders for the present study and, if applicable, for the original study on which the present article is based	13

The STROBE checklist is distributed under the terms of the Creative Commons Attribution License CC-BY. This checklist was completed on 19. February 2023 using https://www.goodreports.org/, a tool made by the EQUATOR Network in collaboration with Penelope.ai

For peer review only
